# Effectiveness of amoxicillin and amoxicillin-clavulanate for the treatment of community-acquired pneumonia in adults and children: a systematic review and meta-analysis

**DOI:** 10.1136/bmjopen-2025-112219

**Published:** 2026-05-07

**Authors:** Lilia Potter-Schwartz, Myo MM Swe, Michael Sharland, Julia Anna Bielicki, Ben S Cooper, Cherry Lim

**Affiliations:** 1 Center for Infectious Disease Modeling and Analysis, Yale School of Public Health, New Haven, Connecticut, USA; 2 Nuffield Department of Medicine, Centre for Tropical Medicine and Global Health, University of Oxford, Oxford, UK; 3 Mahidol Oxford Tropical Medicine Research Unit, Bangkok, Thailand; 4 City St. George’s University of London, London, United Kingdom, London, UK; 5 Paediatric Research Centre, University Children’s Hospital Basel, University of Basel, Basel, Switzerland

**Keywords:** Systematic Review, Antibiotics, Public health

## Abstract

**Abstract:**

**Objectives:**

The aim of this study is to evaluate existing evidence on the effectiveness of amoxicillin and amoxicillin-clavulanate for community-acquired pneumonia in children and adults.

**Design:**

Systematic review and meta-analysis.

**Data sources:**

PubMed, Cochrane Library, Web of Science and Ovid-MEDLINER were searched with no language restrictions through 16 July 2024.

**Eligibility criteria:**

We included studies comparing the effectiveness of amoxicillin or amoxicillin-clavulanate versus other antibiotics or placebo.

**Data extraction and synthesis:**

Only randomised controlled trials comparing amoxicillin or amoxicillin-clavulanate with another antibiotic or placebo with a primary outcome of clinical resolution or clinical failure were eligible for our review. We used random-effects and fixed-effects logistic regression models to estimate the pooled treatment effect size. Heterogeneity of the studies was evaluated using the τ statistic. We performed an unplanned frequentist random-effects network meta-analysis for the indirect comparison between amoxicillin and amoxicillin-clavulanate. The revised Cochrane risk of bias tool for randomised trials was used to assess and categorise studies into low risk of bias, some concerns or high risk of bias.

**Results:**

We extracted data from 44 studies including 45 400 patients. We found no evidence of a differential effect on clinical resolution when comparing amoxicillin with other antibiotics (n=15 trials; pooled OR 0.88; 95% CI 0.56 to 1.38, where >1 favours amoxicillin) or amoxicillin-clavulanate with other antibiotics (n=17; OR 0.89; 95% CI 0.76 to 1.04). Similarly, evidence of difference in clinical failure between amoxicillin and other antibiotics was unclear and unable to rule out clinically important benefits or harms (n=8; OR 0.76; 95% CI 0.55 to 1.06, where <1 favours amoxicillin). We found no evidence of a differential effect on clinical resolution between adults treated with amoxicillin and amoxicillin-clavulanate (n=28; OR 1.04; 95% CI 0.64 to 1.70, where >1 favours amoxicillin-clavulanate). Sixty-three per cent and 29% of amoxicillin and amoxicillin-clavulanate studies, respectively, had low risk of bias according to the Cochrane risk of bias tool for randomised trials.

**Conclusions:**

Current evidence is unclear as to whether amoxicillin or amoxicillin-clavulanate differs from other antibiotics, or from each other, in the treatment of community-acquired pneumonia, owing to the small number of trials and substantial heterogeneity in comparators used across study settings.

**PROSPERO registration number:**

CRD42024568554.

**STRENGTHS AND LIMITATIONS OF THIS STUDY**

- We comprehensively searched multiple databases to ensure inclusion of a maximum number of relevant randomised control trials for our systematic review and meta-analysis.
- We used random-effects and fixed-effects logistic regression models to estimate the pooled treatment effect size across the prespecified primary and secondary outcomes of interest.
- There was an insufficient number of trials to comprehensively adjust for differences in amoxicillin doses and treatment duration in the main analysis.
- The unplanned network meta-analysis was based on trials focusing on amoxicillin or amoxicillin-clavulanate only, thereby potentially excluding other relevant trials that could contribute indirect evidence.

## Introduction

 Lower respiratory infections remain the world’s leading infectious cause of death.^1^ Specifically, pneumonia causes a high global burden of death and morbidity, disproportionately affecting children <5 years of age.^2^ In 2021, there were an estimated 0.5 million (95% UI 0.4 to 0.6) pneumonia deaths in children under five. Community-acquired pneumonia (CAP) is one of the most common causes of childhood hospitalisations in high-income countries and the leading cause of death among children in low- and middle-income countries.^2^,^4^ The empirical antibiotic treatment guidelines of CAP vary by country and can be based on severity scores and local epidemiology.^5^,^7^

Amoxicillin is recommended by WHO as the first-choice antibiotic for adults and children in 10 of the 12 most common primary care infections for all WHO regions, making it a critical ‘Access’ antibiotic. In particular, it is recommended as the first choice for mild/moderate bacterial CAP in adults, with amoxicillin-clavulanate (amoxicillin+clavulanate) as the second-choice antibiotic.^8 9^ Amoxicillin is also the WHO-recommended first-choice treatment for all children with non-severe pneumonia.^8 9^ Similarly, for the WHO AWaRe book, amoxicillin is the first choice for bacterial CAP in adults and children, while amoxicillin-clavulanate is a second-choice recommendation.^8 9^ Improving the evidence base for the use of amoxicillin empirically for CAP could help support its key role in achieving the global United Nations General Assembly antimicrobial resistance target of 70% of overall human antibiotic consumption from the ‘Access’ group by 2030.

There is increasing evidence of the importance of beta-lactam monotherapies, such as amoxicillin, in the treatment of CAP to reduce the use of macrolides and fluoroquinolones due to concerns about potentially increased selection for resistance with the latter Watch or Reserve antibiotics compared with Access antibiotics.^10^ While clinical trials have examined amoxicillin across different settings, studies directly comparing amoxicillin and amoxicillin-clavulanate are lacking. We performed a systematic review of trials of amoxicillin or amoxicillin-clavulanate and evaluated the effectiveness in empirical treatments for CAP between the two regimens and other antibiotics using a meta-analysis.

## Methods

### Search strategy

Our search strategy of randomised trials across four databases used keywords pertinent to the evaluation of amoxicillin and amoxicillin-clavulanate in treatment of CAP. We searched PubMed, Cochrane Library, Web of Science and Ovid-MEDLINER databases using the following search terms: (amoxicillin[Title/Abstract] OR amoxicillin-clavulanate[Title/Abstract] OR amoxicillin/clavulanic acid[Title/Abstract] OR amoxycillin[Title/Abstract] OR hydroxyampicillin[Title/Abstract] OR amoxil[Title/Abstract] OR clamoxyl[Title/Abstract] OR trimox[Title/Abstract] OR wymox[Title/Abstract] OR polymox[Title/Abstract] OR larotid[Title/Abstract] OR augmentin[Title/Abstract]) AND (pneumonia[Title]) NOT (review[Title]).

The full search strategies are presented in the online supplemental material.

Each source was last searched on 16 July 2024, and reference lists of the included articles were screened, inclusive of articles published in any language. Our review followed a prespecified protocol and was registered with the International Prospective Register of Systematic Reviews (PROSPERO registration number: CRD42024568554). We followed the Preferred Reporting Items for Systematic Reviews and Meta-Analyses guidelines.^11^

### Eligibility criteria

Studies were eligible if (i) they were randomised controlled trials (RCTs), (ii) the objectives were to compare amoxicillin or amoxicillin-clavulanate with another antibiotic or placebo for either outpatients or inpatients diagnosed with CAP, (iii) published on or prior to 16 July 2024. Observational studies, qualitative studies, case reports and reviews were excluded. Additionally, studies were excluded for the meta-analysis if patients with other infections besides CAP were randomised, multiple antibiotics were used in the amoxicillin arm (ie, combined treatment of amoxicillin with other antibiotics) or the primary outcome was neither clinical resolution nor clinical failure.

### Study selection

**Figure 1:**
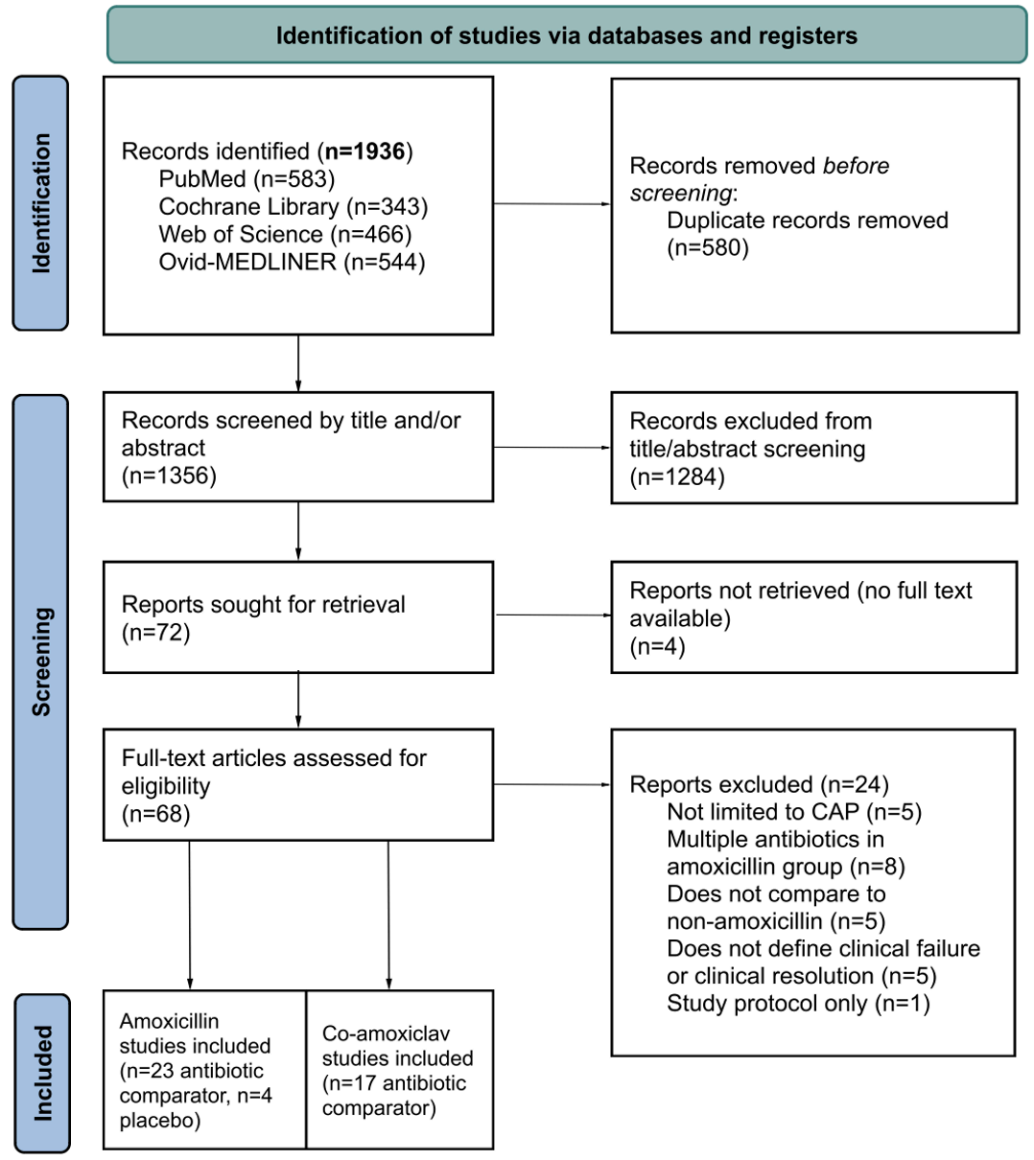
Preferred Reporting Items for Systematic Reviews and Meta-Analyses 2020 flow diagram for systematic reviews. CAP, community-acquired pneumonia.

We first removed duplicated studies, and then screened the titles and abstracts. Two independent reviewers (LP-S, MMMS) performed the screening to select articles for a full review. Any disagreements between the reviewers were resolved by a third reviewer (CL). A full article review was then conducted for each selected study. We recorded the reasons for excluding studies (figure 1; https://github.com/liliapsnh/cap_amoxicillin.git).

### Data extraction

We developed a data extraction spreadsheet with the intended variables. After piloting data extraction for five randomly selected articles meeting inclusion criteria, the spreadsheet was finalised using feedback from four reviewers (LP-S, MMMS, BC, CL). The list of extracted variables included title, first author, journal, PubMed Unique Identifier, Digital Object Identifier, publication year, study type, setting, study blinding, CAP criteria, patient population, geographical region, sample size, primary and secondary clinical outcomes, drug adherence, patients lost to follow-up, adverse events and type of analysis (intention-to-treat or per-protocol). The antibiotic name, dose and route of administration for both the intervention and comparator treatments were also extracted. The primary outcomes extracted from the included trials were clinical resolution and clinical failure, with a secondary outcome of microbiological success (confirmed or presumed bacteriological eradication).

### Risk-of-bias assessment

The revised Cochrane risk of bias tool for randomised trials (RoB2) was used to assess and categorise studies into low risk of bias, some concerns or high risk of bias. The five domains of the RoB2 tool assessed the randomisation process, deviations from intended interventions, missing outcome data, measurement of the outcome and selection of the reported result. Each of these domains was assessed using the Cochrane signalling questions. After answering each of the signalling questions, we used the Cochrane mapping tools to identify the level of risk associated with each study. Two reviewers (LP-S, MMMS) independently evaluated the studies using the RoB2 tool, with a third reviewer (CL) resolving any conflicts that arose.

### Statistical analysis

Additional steps were taken to ensure that participants from the three-arm trials were not double counted in the pooled analyses. As recommended by the Cochrane Handbook, for the three-arm studies that compared different doses of the same antibiotic, we combined these into one data point per study, thereby transforming them into pseudo two-arm studies.^12^ For the three-arm studies that compared two different antibiotics, we halved the amoxicillin-clavulanate samples to ensure the participants were not double counted.^12^

We used the *meta* package in R (V.4.4.0) to perform the meta-analyses.^13^ The *meta* package was used to calculate pooled ORs and generate forest plots. We evaluated the effect of amoxicillin against alternative antibiotics on outcomes using a random-effects logistic regression model, assuming that each study had its own magnitude of effect, to include between-study variability into the estimation of the overall treatment effects. We used a fixed-effects model for the trials comparing amoxicillin with a placebo, given that there was no variation in the dosage or frequency of the comparators across these trials. Additionally, we performed a sensitivity analysis with fixed-effects models using the Hartung-Knapp-Sidik-Jonkman method. The effect sizes were reported as a pooled OR.

Separate models were fitted on data from studies that included amoxicillin-clavulanate in one of the treatment arms. Two primary outcomes, which are clinical resolution and treatment failure, were analysed in separate models. Secondary outcome analysis for bacterial eradication was also performed. We performed subgroup analyses by age group (<5 and >5 years), disease severity, antibiotic class and dose, following the prespecified protocol. We performed preplanned subgroup analyses according to the protocol, and pooled ORs with corresponding CIs were calculated for each analysis.

We conducted a second sensitivity analysis, where we combined the ‘clinical failure’ and ‘clinical resolution’ studies comparing amoxicillin with an alternative antibiotic treatment by considering patients in the ‘clinical failure’ trials who did not reach the clinical failure end point to have achieved clinical resolution, excluding patients lost to follow-up.

We also evaluated the effect of amoxicillin compared with other antibiotic treatments on outcomes using fixed-effects logistic regression models as a third sensitivity analysis, assuming that the underlying magnitude of the effect of amoxicillin on the outcome was the same across the studies included in the meta-analysis.

Finally, we conducted an unplanned frequentist random-effects network meta-analysis to compare amoxicillin and amoxicillin-clavulanate, assuming that each study had its own true effect. Network meta-analysis relies on the transitivity assumption—that the distribution of effect modifiers is similar across treatment comparisons. To mitigate the risk of violating this assumption, we restricted the analysis to trials involving adult patients only. Furthermore, we assessed the transitivity assumption by examining the distribution of potential effect modifiers—age, setting, dosage, duration and CAP severity—across treatment comparisons. These potential effect modifiers were chosen to ensure there were no significant differences that could influence the comparability of treatment effects across the network. In addition, net heat plots and the node-splitting method were used to evaluate agreement between direct and indirect evidence in the network (ie, the consistency assumption). The *netmeta* package was used for the network plots and network meta-analysis.

Heterogeneity of the studies was evaluated using the τ statistic. The statistical heterogeneity expressed with τ conveys the degree of heterogeneity across the included studies.^12^

### Patient and public involvement

Patients and/or the public were not involved in the design, or conduct, or reporting, or dissemination plans of this research.

## Results

### Study characteristics

We identified 1936 records across four databases (PubMed, Cochrane Library, Web of Science and Ovid-MEDLINER) and after removing duplicates, we screened 1356 abstracts, of which 68 articles underwent full-text assessment. Of the 68 articles, 24 were excluded for various reasons, including five paediatric CAP studies that did not report the specified primary outcome (figure 1). Instead, these studies reported alternative outcomes such as time to clinical improvement, time to defervescence and resistance patterns of bacterial pathogens isolated from tracheal secretions.

There were 27 studies on amoxicillin and 17 studies on amoxicillin-clavulanate that met the eligibility criteria and were included in the final review and meta-analysis (figure 1, online supplemental figure 1). Of the 27 RCTs comparing amoxicillin with other antibiotics or placebo, 15^14^,^28^ and 12 reported primary outcomes for clinical resolution and clinical failure, respectively, for patients with CAP. The 17^29^,^45^ studies comparing amoxicillin-clavulanate with other antibiotics reported a primary outcome of clinical resolution.

The amoxicillin and amoxicillin-clavulanate RCTs included in the review represented 39 968 and 5432 patients, respectively, ranging from 32 to 15 662 patients per trial (online supplemental table 1). There were six amoxicillin-clavulanate three-arm studies that compared either two different dosages of the same antibiotic (levofloxacin, cefditoren) or two other different antibiotics with amoxicillin-clavulanate within the same study. Four of the amoxicillin studies were placebo-controlled, and all four trials were on patients <5 years of age with non-severe diagnoses. The dose and duration of amoxicillin and amoxicillin-clavulanate treatment varied by study, as well as the clinical severity, country and study population (https://github.com/liliapsnh/cap_amoxicillin.git). The duration of amoxicillin ranged from 2 to 14 days, and the dosage ranged from 1 to 4 g/day for adult patients.

### Comparator antibiotics

The trials included in this systematic review compared amoxicillin with 15 different antibiotics and amoxicillin-clavulanate with 13 different antibiotics. By antibiotic class, the trials included treatment arms that compared amoxicillin with beta-lactams (n=8), macrolides (n=3), quinolones (n=6) and sulfonamides (n=6); and amoxicillin-clavulanate with beta-lactams (n=10), macrolides (n=7) and quinolones (n=7). The six studies with sulfonamides all compared oral amoxicillin with oral co-trimoxazole in study populations of children <5 years of age with non-severe CAP. After co-trimoxazole, the most frequent pairs of comparator antibiotics included amoxicillin-clavulanate to ceftriaxone (n=4), amoxicillin-clavulanate to cefuroxime (n=3), amoxicillin-clavulanate to erythromycin (n=3) and amoxicillin to benzylpenicillin (n=3).

### Primary and secondary outcomes

**Figure 2:**
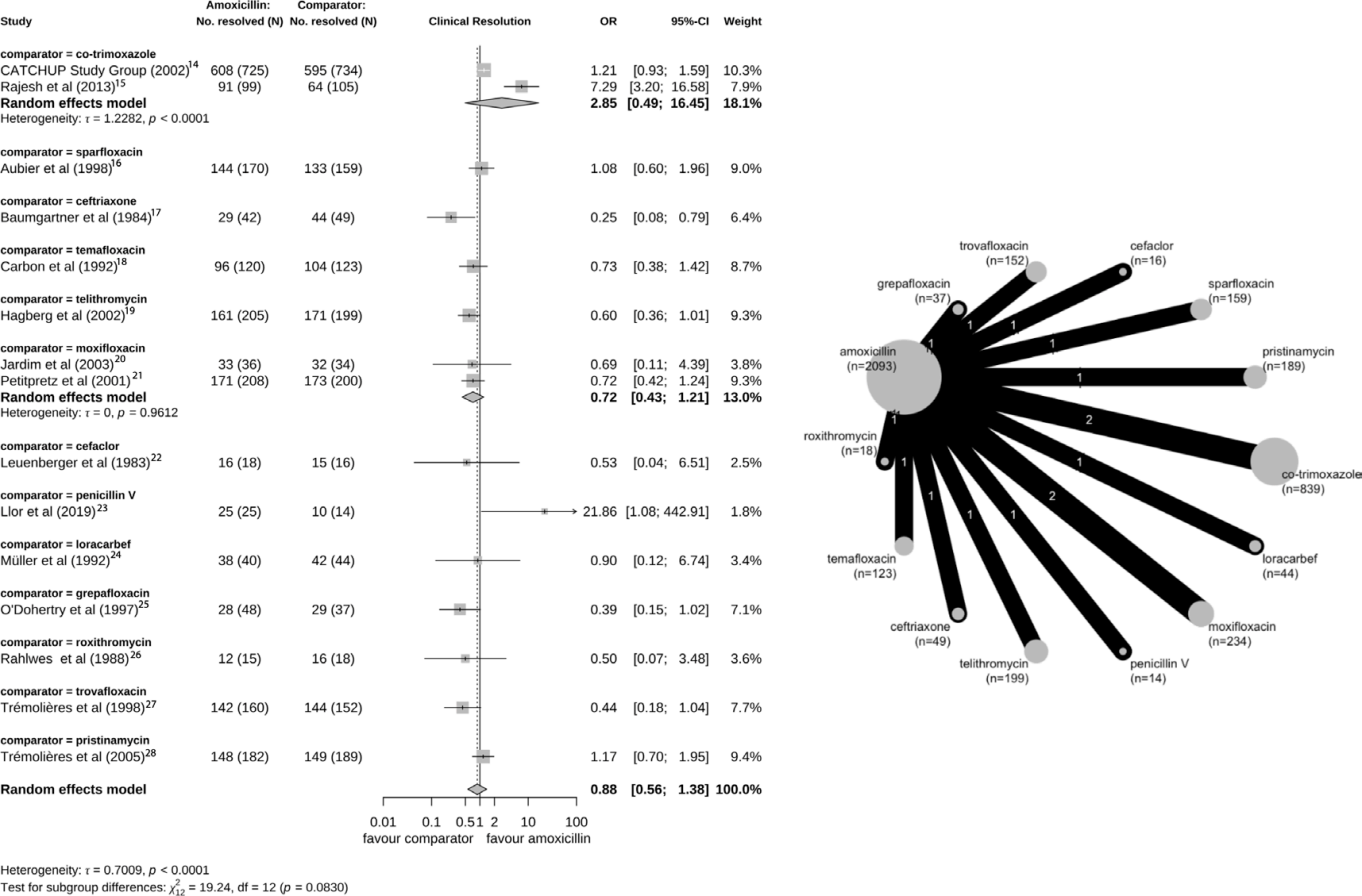
Forest plot and network graph for the 15 studies with a primary outcome of clinical resolution comparing amoxicillin with another antibiotic treatment.^14^,^28^ Two studies (CSG, Rajesh) consider a patient population <5 years of age. A larger area of the circle in the network diagram reflects a higher number of papers using the antibiotic in one of the treatment arms.

**Figure 3:**
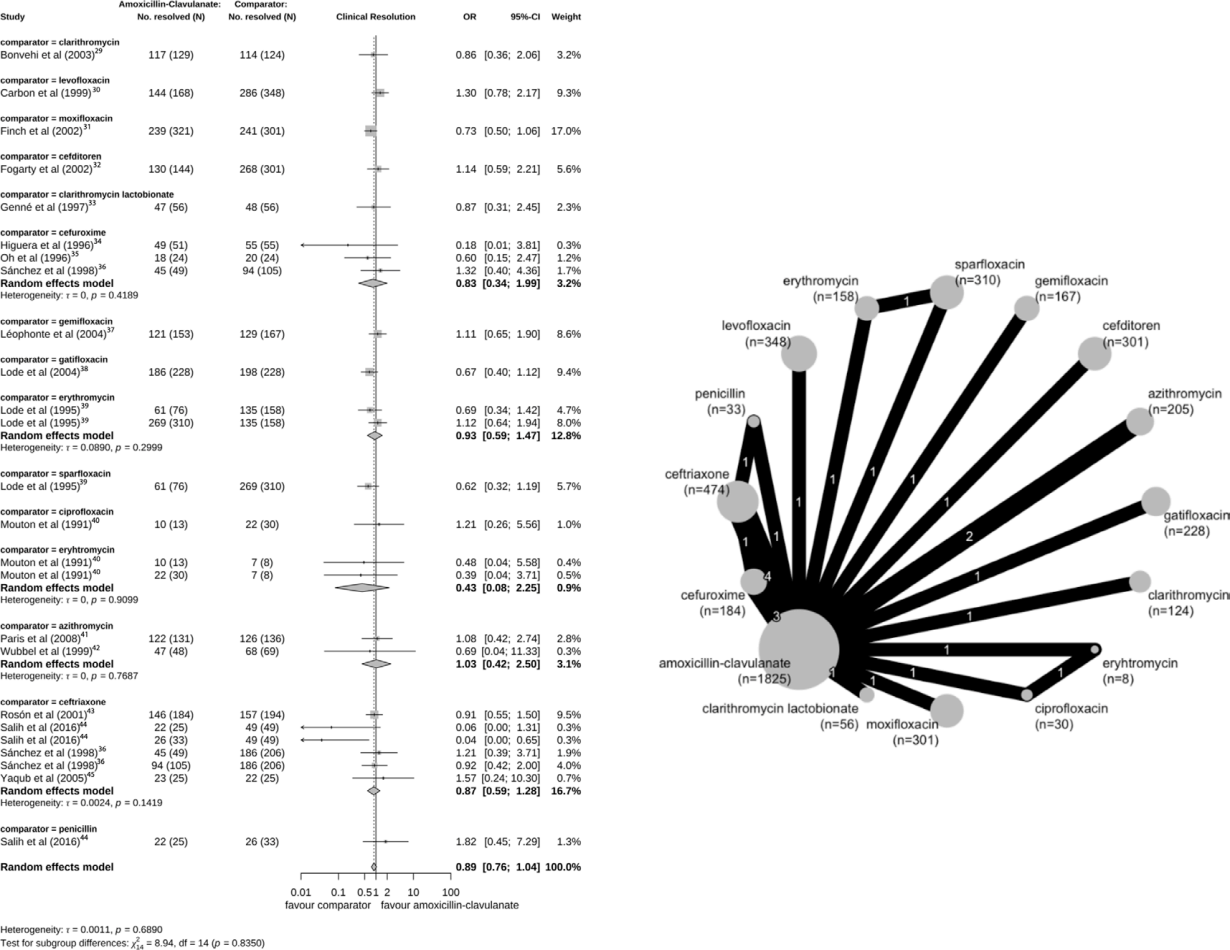
Forest plot and network graph for the 17 studies with a primary outcome of clinical resolution comparing amoxicillin-clavulanate with another antibiotic treatment.^29^,^45^ Two studies (Salih, Wubbel) consider a patient population <5 years of age.

The definition of clinical resolution used in the trials was resolution of symptoms (29 studies) or no requirement for further antibiotic therapy (three studies). Five amoxicillin and three amoxicillin-clavulanate studies included confirmation from chest X-ray results at test of cure visits. The clinical resolution outcome was examined at various time points ranging from day 5 of treatment to 25 days post-therapy. The random effects model did not find strong evidence of differences in the odds of clinical resolution between amoxicillin treatment compared with other antibiotic treatment (n=15 trials; pooled OR 0.88; 95% CI 0.56 to 1.38, where >1 favours amoxicillin; τ=0.70) (figure 2) and amoxicillin-clavulanate compared with other antibiotics (n=17; OR 0.89, 95% CI 0.76 to 1.04; τ=0) (figure 3). The findings from the sensitivity analysis using the Hartung-Knapp-Sidik-Jonkman method showed similar patterns with wider CIs (amoxicillin clinical resolution compared with other treatment: OR 0.88; 95% CI 0.53 to 1.47; τ=0.70 and amoxicillin-clavulanate resolution compared with other treatment: OR 0.89; 95% CI 0.76 to 1.03; τ=0).

**Figure 4:**
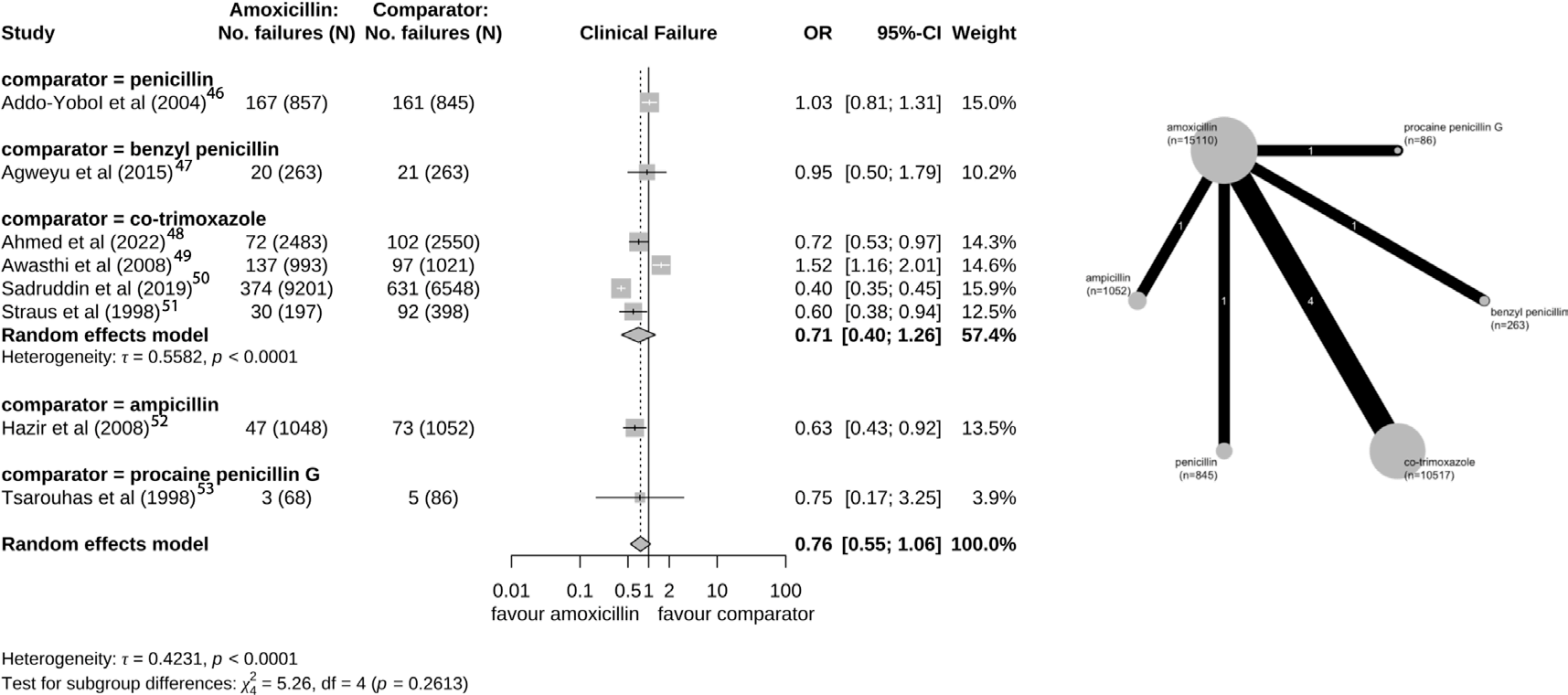
Forest plot and network graph for the eight studies with a primary outcome of clinical failure comparing amoxicillin with another antibiotic (non-placebo).^46^,^53^ One study (Tsarouhas et al (1998))^53^ considers a patient population of >5 years of age.

Twelve of the 27 studies with an amoxicillin treatment arm analysed clinical failure as the primary outcome. In 10 studies, the definition of clinical failure was persistence of symptoms, while in two studies it was the requirement to change antibiotic regimen. The clinical failure outcome was examined at various time points ranging from 48 hours to 14 days after starting the treatment regimen. There were eight trials 46,52 comparing clinical failure in amoxicillin with another antibiotic treatment (non-placebo trial), with large heterogeneity.^53^ The reported OR from those trials ranged from 0.66 (95% CI 0.5 to 0.86) to 2.52 (95% CI 2.21 to 2.87) with a pooled OR of 0.76 (95% CI 0.55 to 1.06; τ=0.42), where <1 favours amoxicillin (figure 4).

Thirteen studies reported bacteriological eradication as a secondary outcome for patients with available microbiological samples. The pooled OR for the studies comparing amoxicillin with alternative antibiotics was 0.53, where >1 favours amoxicillin (95% CI 0.32 to 0.87; τ=0) (online supplemental figure 2) and for studies comparing amoxicillin-clavulanate with alternative antibiotics, it was 0.80 (95% CI 0.50 to 1.27; τ=0) (online supplemental figure 3).

The sensitivity analysis that combined the eight clinical failure and 15 clinical resolution studies comparing amoxicillin with an alternative antibiotic treatment yielded an OR of 1.06 (95% CI 0.79 to 1.42; τ=0.57), where >1 favours amoxicillin for clinical resolution. And the fixed-effects sensitivity analysis, under the assumption that there was a true underlying effect across all studies, yielded narrower CIs (online supplemental figures 4 and 5).

**Figure 5:**
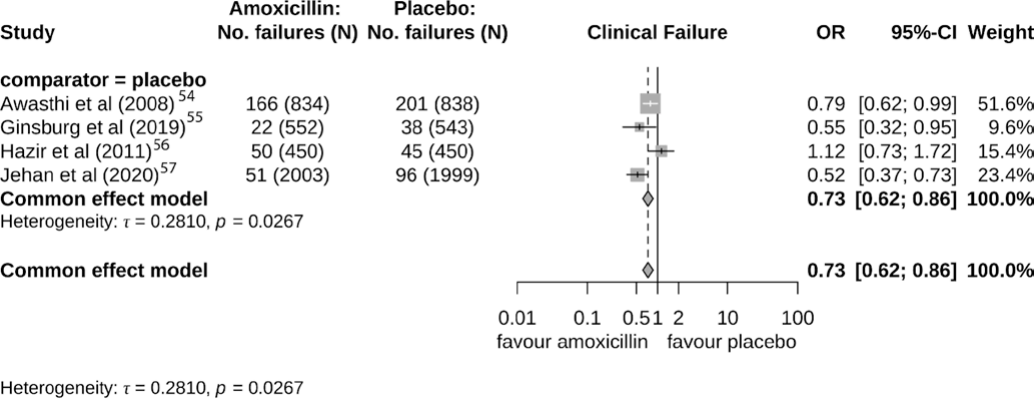
Forest plot for fixed-effects model of clinical failure and placebo-comparator only.

A clinical benefit of amoxicillin compared with placebo in four studies^54^,^57^ was observed with an estimated pooled OR of 0.73 (95% CI 0.62 to 0.86; τ=0.28) (figure 5).

### Subgroup analyses

We stratified the included studies by clinical outcome, age group, antibiotic class and amoxicillin dose. Thirteen (48%) of the amoxicillin and two (12%) of the amoxicillin-clavulanate studies assessed patient populations <5 years of age. We observed similar patterns across subgroup analyses by antibiotic class (online supplemental figures 6–8) and amoxicillin or amoxicillin-clavulanate dose (online supplemental figures 9–14).

### Risk-of-bias assessment

The distribution of overall risk of bias scores among the amoxicillin studies was as follows: 63% (n=17) were classified as low risk, 26% (n=7) had some concerns and 11% (n=3) were high risk (online supplemental figure 15). Corresponding numbers for amoxicillin-clavulanate studies were: 29% (n=5) low risk, 47% (n=8) some concerns and 24% (n=4) high risk (online supplemental figure 15). The most common reasons for concerns of bias included limited information about the randomisation process, a high proportion of patients (>10%) lost to follow-up, and failure to report results on an intention-to-treat basis.

### Unplanned network meta-analysis

There were 28 studies evaluating adult patients with an outcome of clinical resolution. Our unplanned network meta-analysis on those studies found no evidence of differential outcomes for adults treated with amoxicillin versus those treated with amoxicillin-clavulanate (n=28; OR 1.04; 95% CI 0.64 to 1.70, where>1 favours amoxicillin-clavulanate) (online supplemental figure 16).

The global Q score for inconsistency was 7.36 (p=0.195), and the only comparisons that were inconsistent were ceftriaxone versus amoxicillin (p=0.021) and ceftriaxone versus amoxicillin-clavulanate (p=0.018) (online supplemental table 2, online supplemental figure 17). However, the tests have limited power as the network of the 28 studies was sparse. We found no clear evidence of violations of the transitivity assumption when comparing age, setting, dosage, duration and severity of studies across the comparisons (online supplemental table 3). The mean age across all amoxicillin or amoxicillin-clavulanate arms in the 28 studies was 51.5 years (SD 7.83), and the average duration was approximately 8.96 years (SD1.94). However, the number of studies per comparison was small.

## Discussion

Our systematic review and meta-analysis of the 44 included trials did not find clear evidence of differential effectiveness of either amoxicillin or amoxicillin-clavulanate compared with other antibiotic treatments, with CIs unable to rule out clinically significant benefits or harms. The four identified placebo trials for amoxicillin, performed in India, Malawi and Pakistan, highlighted the effectiveness of amoxicillin among children with non-severe CAP in low- and middle-income countries and pointed to the importance of ensuring sustainable access to amoxicillin.^24 28 31 33^

Since finalising our results, we have conducted an additional search to identify any trials that may have been published between 16 July 2024 and 20 January 2026. This search yielded one trial published by Qin *et al*, which found amoxicillin-clavulanate intravenous infusion for 7–14 days was non-inferior to ampicillin-sulbactam in terms of clinical resolution and microbiological efficacy in the treatment of adult patients with CAP.^58^

In comparison with our findings, a population-based emulated target trial that compared 25 332 cases of paediatric pneumonia in Sweden showed higher risk of treatment failure in the penicillin V treatment group compared with the amoxicillin treatment group (7.7% vs 4.7%, adjusted OR 1.76, 95% CI 1.54 to 2.00).^59^ Recent clinical trials have also focused on effective duration and dose of amoxicillin for community-acquired paediatric pneumonia, suggesting similar treatment outcomes between low-dose and high-dose amoxicillin.^60^,^63^

Through our systematic review and meta-analysis, we found unclear evidence of difference in effectiveness between amoxicillin-clavulanate and amoxicillin. In comparison, a retrospective observational study of electronic health records found no evidence of a difference in mortality between the two treatments for CAP.^64^ Similarly, a multicentre randomised trial conducted in seven hospitals in the Netherlands showed narrow-spectrum treatment for non-intensive care unit CAP was non-inferior to broad-spectrum treatment.^65^ However, it is important to note that these findings are likely impacted by local rates of resistance for each given setting.

Limitations of our study included an insufficient number of trials to comprehensively adjust for differences in amoxicillin doses and treatment duration in the main analysis. There was also inconsistent reporting of microbiological cure rates in the included trials and heterogeneity in the type of comparator antibiotics used across the trials identified, which could explain the observed variations in the treatment effect. Our unplanned network meta-analysis was based on trials focusing on amoxicillin or amoxicillin-clavulanate only. There may be other relevant trials that could contribute indirect evidence for the comparison between amoxicillin and amoxicillin-clavulanate that were not included in this analysis. Future studies would be needed to strengthen the evidence for the recommendations for the choice between those two antibiotics for empiric treatment in CAP.

## Conclusion

In conclusion, we found no clear evidence of differences in clinical outcomes with amoxicillin compared with either amoxicillin-clavulanate or other antibiotics used for the treatment of CAP. While large uncertainties exist around the pooled estimates, the available evidence is consistent with the WHO AWaRe book recommendation to use amoxicillin as the first-choice antibiotic for the treatment of CAP in adults and children. Moreover, our findings emphasise the need for further RCTs comparing treatment options across populations and settings, including regions with differing levels of resistance.

## Supplementary material

10.1136/bmjopen-2025-112219 Supplementary file 1

## Data Availability

All data relevant to the study are included in the article or uploaded as supplementary information.
