# Supplementary material for: Effectiveness of amoxicillin and amoxicillin-clavulanate for the treatment of community-acquired pneumonia in adults and children: a systematic review and meta-analysis
Source: BMJ Open. 2026 May 7;16(5):e112219. doi: 10.1136/bmjopen-2025-112219 (PMC13157766; doi:10.1136/bmjopen-2025-112219)
Supplement: Supplementary file 1 [file bmjopen-16-5-s001.pdf]

Supplementary Information

Search Strategy

1. **PubMed**
   ((amoxicillin[Title/Abstract] OR amoxicillin-clavulanate[Title/Abstract] OR amoxicillin/clavulanic acid[Title/Abstract] OR amoxycillin[Title/Abstract] OR hydroxyampicillin[Title/Abstract] OR amoxil[Title/Abstract] OR clamoxyl[Title/Abstract] OR trimox[Title/Abstract] OR wymox[Title/Abstract] OR polymox[Title/Abstract] OR larotid[Title/Abstract] OR augmentin[Title/Abstract]) AND (pneumonia[Title])
   NOT (review[Title]))
2. **Cochrane Library**
   (amoxicillin OR amoxicillin-clavulanate OR amoxicillin-clavulanic acid OR amoxycillin OR hydroxyampicillin OR amoxil OR clamoxyl OR trimox OR wymox OR polymox OR larotid OR augmentin):ti,ab,kw
   AND (pneumonia):ti
   NOT (review):ti
3. **Web of Science**
   AB=(amoxicillin OR amoxicillin-clavulanate OR amoxicillin-clavulanic acid OR amoxycillin OR hydroxyampicillin OR amoxil OR clamoxyl OR trimox OR wymox OR polymox OR larotid OR augmentin) AND TI=(pneumonia) NOT TI=(review)
4. **Ovid-MEDLINER**
   (amoxicillin or amoxicillin-clavulanate or amoxicillin-clavulanic acid or amoxycillin or hydroxyampicillin or amoxil or clamoxyl or trimox or wymox or polymox or larotid or augmentin).ab. and pneumonia.ti. not review.ti.

Supplementary Table 1. A. Characteristics of the 27 included studies with an amoxicillin arm.

Author (Publication Year)	Country	Study Design	Setting	Patients	Sample Size	Daily dose, Route, and Duration of Amoxicillin	Daily dose, Route, and Duration of Comparator(s)	Extracted Outcomes
CATCHUP Study Group (2002)[14]	Pakistan	Randomized controlled, double blind, multicentre	Outpatient	Children <5 with non-severe CAP	1471	Oral amoxicillin: 50 mg/kg, 7d	Oral co-trimoxazole: 8/40mg/kg, 7d	Clinical resolution

Addo-YoboI *et al* (2004)[46]	8 countries in Africa, Asia, and South America	multicentre, randomized, open-label equivalency	Inpatient	Children <5 with severe CAP	1702	Oral amoxicillin: 45 mg/kg, 48h min.	Injectable penicillin: 200000 IU/kg, 48h min.	Clinical failure
Agweyu *et al* (2015)[47]	Kenya	Open-label, multicentre, randomized controlled noninferiority	Inpatient	Children <5 with severe CAP	521	Oral amoxicillin: 80-90 mg/kg, 48h minimum	i.v./i.m. benzyl penicillin: 200000 IU/kg, 48h min.	Clinical failure
Ahmed *et al* (2022)[48]	Pakistan	Cluster-randomized, unblinded, community-based	Outpatient	Children <5 with fast-breathing CAP	4984	Oral amoxicillin: 250mg/5ml, 3d	Oral co-trimoxazole: 200mg/40mg per 5ml, 5d	Clinical failure
Aubier *et al* (1998)[16]	46 centres in France, South Africa, and Switzerland	Randomized, double-blind, multicentre	Inpatient	Adults hospitalised with CAP	329	Oral amoxicillin: 3000g, 10d	Oral sparfloxacin: 400mg loading, 200mg, 10d	Clinical resolution
Awasthi *et al* (2008)[54]	India	Multi-centric, randomized placebo controlled double blind clinical	Outpatient	Children <5 with non-severe CAP	1671	Oral amoxicillin: 31-54mg/kg, 3d	Placebo: 3d	Clinical resolution
Awasthi *et al* (2008)[49]	India	Cluster randomized, open labelled	Outpatient	Children <5 with non-severe CAP	2009	Oral amoxicillin: 31-51 mg/kg, 3d	Oral co-trimoxazole: 7-11mg/kg, 5d	Clinical failure
Baumgartner *et al* (1984)[17]	Switzerland	Randomized, controlled	Inpatient	Adults with acute CAP	1984	i.v. amoxicillin: 4g, duration varied	i.v. ceftriaxone, 2g, duration varied	Clinical resolution
Carbon *et al* (1992)[18]	France	Double-blind, multicentre	Inpatient	Adults hospitalised with CAP	246	Oral amoxicillin: 1000mg, 10d mean	Oral temafloxacin: 1200mg, 10d mean	Clinical failure

Ginsburg *et al* (2019)[55]	Malawi	Double-blind, randomized clinical noninferiority	Outpatient	Children <5 with non-severe fast-breathing CAP	1126	Oral amoxicillin: 500-1500mg, 3d	Placebo: 3d	Clinical failure
Hagberg *et al* (2002)[19]	13 countries in South America, Oceania, Europe and Africa	Randomized, double-blind	Mixed	Adults with CAP	404	Oral amoxicillin: 3000mg, 10d	Oral telithromycin: 800mg, 10d	Clinical resolution
Hazir *et al* (2008)[52]	Pakistan	Randomized, open-label, equivalency	Mixed	Children <5 with severe CAP	2037	Oral amoxicillin: 80-90 mg/kg, 5d	Parenteral ampicillin: 100mg/kg, 3d	Clinical failure
Hazir *et al* (2011)[56]	Pakistan	Double-blind, randomized, equivalence	Inpatient	Children <5 with non-severe CAP	873	Oral amoxicillin: 45 mg/kg, 3d	Placebo: 3d	Clinical failure
Jardim *et al* (2003)[20]	Latin America	Prospective, multicentre, multinational, controlled, randomized, double-blind	Mixed	Adults with CAP	70	Oral amoxicillin: 1500mg, 10d maximum	Oral moxifloxacin: 400mg, 10d	Clinical resolution, microbiologic cure
Jehan *et al* (2020)[57]	Pakistan	Double-blind, randomized, placebo-controlled noninferiority	Outpatient	Children <5 with non-severe CAP	4002	Oral amoxicillin: 1000-3000mg, 3d	Placebo: 3d	Clinical failure
Leuenberger *et al* (1983)[22]	Switzerland	Randomized double blind controlled	Inpatient	Adults with CAP	34	Oral amoxicillin: 1500mg, 7d	Oral cefaclor: 2250mg, 7d	Clinical resolution
Llor *et al* (2019)[23]	Spain	multicentre, parallel, double-blind, controlled, randomized	Outpatient	Adults with CAP	43	Oral amoxicillin: 3000mg, 10d	Oral penicillin: 1.6 million units, 10d	Clinical resolution

Müller *et al* (1992)[24]	Canada, Europe, South Africa	Double-blind, randomized, parallel treatment	Outpatient	Adults with CAP	336	Oral amoxicillin: 1500mg, 10-14d	Oral loracarbef: 800mg, 10-14d	Clinical resolution
O'Dohertry *et al* (1997)[25]	UK and Ireland	Randomized, multicentre, double-blind, double-dummy	Outpatient	Adults with CAP	264	Oral amoxicillin: 1500mg, 7-10d	Oral grepafloxacin: 600mg, 7-10d	Clinical resolution
Petitpretz *et al* (2001)[21]	South America, Europe, Asia, North America and South Africa	Multinational, multicentre, double-blind, randomized	Mixed	Adults with CAP	408	Oral amoxicillin: 3000mg, 10d	Oral moxifloxacin: 400mg, 10d	Clinical resolution
Rahlwes *et al* (1988)[26]	Germany	Prospective, randomized, comparative	Inpatient	Adults with CAP	33	Oral amoxicillin: 2250mg, 9.4d mean	Roxithromycin: 300mg, 9.3d mean	Clinical resolution
Rajesh *et al* (2013)[15]	India	Randomized, controlled	Outpatient	Children <5 with non-severe CAP	204	Oral amoxicillin: 40mg/kg, 5d	Co-trimoxazole: 8mg/kg trimethoprim, 5d	Clinical resolution
Sadruddin *et al* (2019)[50]	Pakistan	Unblinded, cluster-randomized, controlled-equivalency	Outpatient	Children <5 with fast-breathing CAP	15662	Oral amoxicillin: 50mg/kg, 3d	Oral co-trimoxazole: 8/40mg/kg, 5d	Clinical failure
Straus *et al* (1998)[51]	Pakistan	Randomized, controlled	Inpatient	Children <5 with non-severe or severe CAP	595	Oral amoxicillin: 45mg/kg, 3-5d	Co-trimoxazole: 80mg/8mg /kg, 3-5d	Clinical failure
Trémolières *et al* (1998)[27]	Europe, South Africa, and Costa Rica	Randomized, multicentre, double-blind study	Mixed	Adults with CAP	312	Oral amoxicillin: 3000g, 7-10d	Oral trovafloxacin: 200mg, 7-10d	Clinical resolution, microbiologic cure

Trémolières *et al* (2005)[28]	France and Tunisia	Multinational, randomized, double blind, double dummy, non-inferiority	Inpatient	Adults with CAP	371	Oral amoxicillin: 3000g, 7-10d	Oral pristinamycin, 3000mg, 7-10d	Clinical resolution
Tsarouhas *et al* (1998)[53]	US	Prospective, randomized, evaluator-blinded	Outpatient	Children (6m-18y) with CAP	170	Oral amoxicillin: 50mg/kg, 2d	i.m. procaine penicillin G: 50,000 units/kg	Clinical failure

Supplementary Table 1. B. Characteristics of the 17 included studies with an amoxicillin-clavulanate arm.

**Author (Publication Year)**	**Country**	**Study Design**	**Setting**	**Patients**	**Sample Size**	**Daily dose, Route, and Duration of Amoxicillin**	**Daily dose, Route, and Duration of Comparator(s)**	**Extracted Outcomes**
Bonvehi *et al* (2003)[29]	45 sites in Argentina, Italy, Mexico, South Africa, Spain, Turkey	Prospective, randomized, investigator-blinded, multicentre	Outpatient	Patients ≥12 with CAP	327	Oral amoxicillin-clavulanate: 1550/250mg, 7d	Oral clarithromycin, 1000mg, 7d	Clinical resolution, microbiologic cure
Carbon *et al* (1999)[30]	50 centres in Argentina, Europe, South Africa	Double-blind, randomized, three-arm parallel, multicentre	Mixed	Adults with mild-to-moderate CAP	516	Oral amoxicillin-clavulanate: 1875mg, 7-10d	Oral levofloxacin: 500mg or 1000mg, 7-10d	Clinical resolution, microbiologic cure
Finch *et al* (2002)[31]	10 countries in Europe, Africa and Asia	Multinational, multicentre with a randomized, open, parallel group design	Inpatient	Adults with CAP	538	i.v./oral amoxicillin-clavulanate: oral dose of 1875g, 7-14d	i.v./oral moxifloxacin: oral dose of 400mg, 7-14d	Clinical resolution, microbiologic cure
Fogarty *et al* (2002)[32]	US	multicentre, prospective, randomized, investigator-blinded, parallel-group	Inpatient	Patients ≥12 with CAP	802	Oral amoxicillin-clavulanate: 1750/250mg, 14d	Oral cefditoren: 400mg or 800mg, 14d	Clinical resolution, microbiologic cure

Genné *et al* (1997)[33]	Switzerland	Prospective, randomized, controlled	Inpatient	Adults with CAP	112	i.v./oral amoxicillin-clavulanate: oral dose of 1875g, 10d min.	i.v./oral clarithromycin lactobionate: oral dose of 1000mg, 10d min.	Clinical resolution
Higuera *et al* (1996)[34]	US and Latin America	multicentre, investigator-blinded	Outpatient	Patients ≥12 with CAP	168	Oral amoxicillin-clavulanate: 1500/375mg, 10d median	Oral cefuroxime axetil: 1000mg, 10d mean	Clinical resolution, microbiologic cure
Léophonte *et al* (2004)[37]	102 centres in France, Poland and South Africa	Randomized, multicentre, double-blind, double-dummy, parallel group Phase III	Mixed	Adults with CAP	254	Oral amoxicillin-clavulanate: 3000/375mg, 10d	Oral gemifloxacin: 320mg, 7d	Clinical resolution, microbiologic cure
Lode *et al* (1995)[39]	9 countries in Europe and Israel	Double-blind, randomized, parallel group	Mixed	Adults with CAP	808	Oral amoxicillin-clavulanate: 1500/375mg, 9-10d mean	Oral sparfloxacin: 400mg loading dose, 200mg, 9-10d mean<br>Oral erythromycin: 2000mg, 9-10d mean	Clinical resolution
Lode *et al* (2004)[38]	16 countries in Europe	Double-blind, double-dummy, multicentre, multinational, parallel-group	Inpatient	Adults with CAP	462	Oral amoxicillin-clavulanate: 1500/375mg, 5-10d	Oral gatifloxacin: 400mg, 5-10d	Clinical resolution
Mouton *et al* (1991)[40]	12 centres in France	Randomized, controlled, multi-centre	Inpatient	Adults with CAP requiring hospitalisation	63	Oral amoxicillin-clavulanate: 2000mg, 5d min.	Oral ciprofloxacin: 1500mg, 5d min.<br>Oral erythromycin: 3000mg, 5d min.	Clinical resolution
Oh *et al* (1996)[35]	Singapore	Randomized trial	Inpatient	Adults with CAP	48	i.v./oral amoxicillin-clavulanate: oral dose of 1500/750mg, 7d min.	i.v./oral cefuroxime: oral dose of 500mg, 7d min.	Clinical resolution

Paris *et al* (2008)[41]	Italy	Randomized, open-label, non-inferiority	Outpatient	Adults with CAP	267	Oral amoxicillin-clavulanate: 1750/250mg, 7d	Oral azithromycin: 1000mg, 3d	Clinical resolution
Rosón *et al* (2001)[43]	Spain	Comparative, prospective, randomized trial	Inpatient	Adults with CAP	378	i.v./oral amoxicillin-clavulanate, oral dose of 3000/375mg, 10.9d mean	i.v./i.m. ceftriaxone: 1000mg, 10.1d mean	Clinical resolution
Salih *et al* (2016)[44]	Sudan	Prospective case control, randomized sampling	Inpatient	Children <60 months with severe CAP	83	Amoxicillin-clavulanate	Penicillin, Ceftriaxone	Clinical resolution
Sánchez *et al* (1998)[36]	Spain	Prospective, comparative	Inpatient	Patients ≥12 with moderate-to-severe CAP	409	i.v./oral amoxicillin-clavulanate: 2625mg oral, 12d total	i.v./oral cefuroxime: 1500g, 12d total i.v./i.m. ceftriaxone: 2g i.v., 5d i.v.	Clinical resolution
Wubbel *et al* (1999)[42]	US	Prospective, randomized, unblinded	Outpatient	Children <5 with CAP	147	Oral amoxicillin-clavulanate: 40mg/kg, 10d	Azithromycin: 10mg/kg d1, 5mg/kg 4d	Clinical resolution
Yaqub *et al* (2005)[45]	Pakistan	Randomized, parallel group comparative	Inpatient	Adults with CAP requiring i.v. antibiotics	50	i.v./oral amoxicillin-clavulanate: 1875mg oral, 7d	Parenteral ceftriaxone: 1-2g, 7d	Clinical resolution

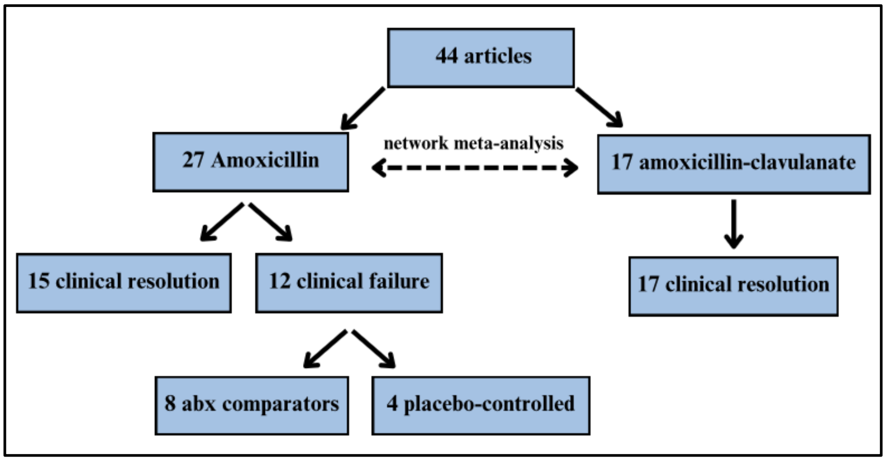

Supplementary Figure 1. Flow chart summary of included articles and primary outcomes.

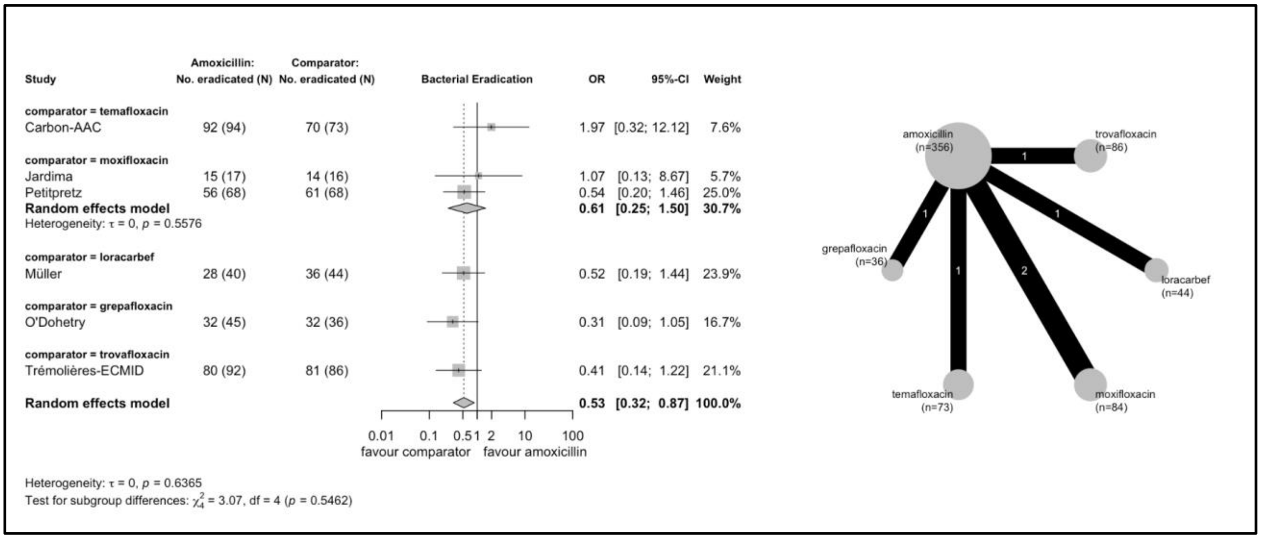

Supplementary Figure 2. Forest plot and network graph for six studies with a secondary outcome of presumed or confirmed bacterial eradication comparing amoxicillin to another treatment.

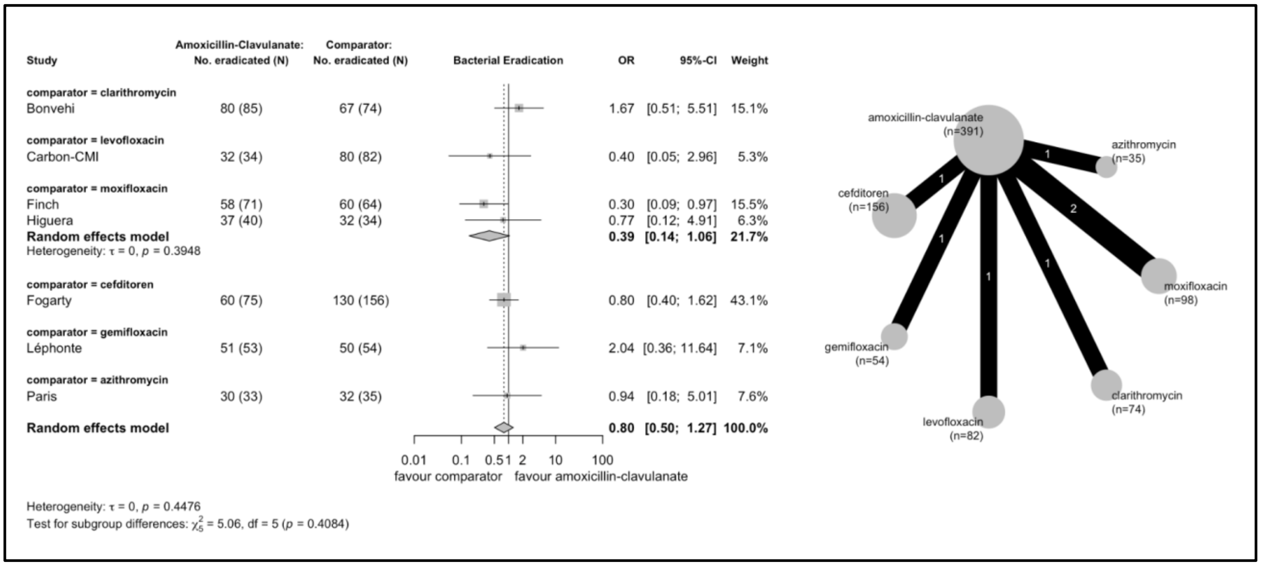

Supplementary Figure 3. Forest plot and network graph for seven studies with a secondary outcome of presumed or confirmed bacterial eradication comparing amoxicillin-clavulanate to another treatment.

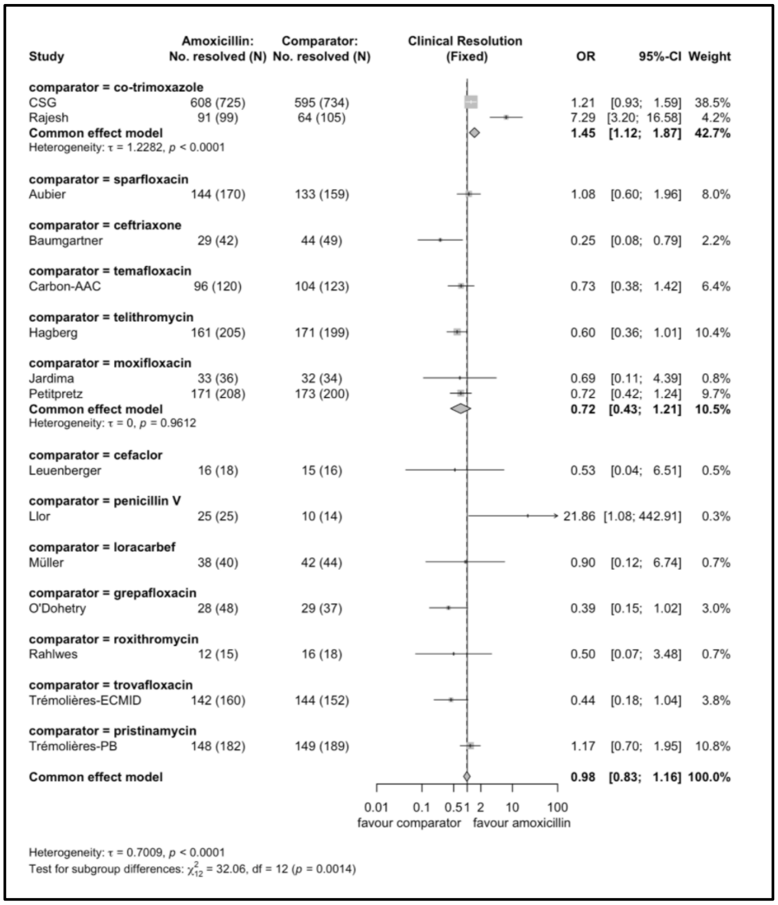

Supplementary Figure 4. Forest plot for fixed effects model of clinical resolution, amoxicillin only.

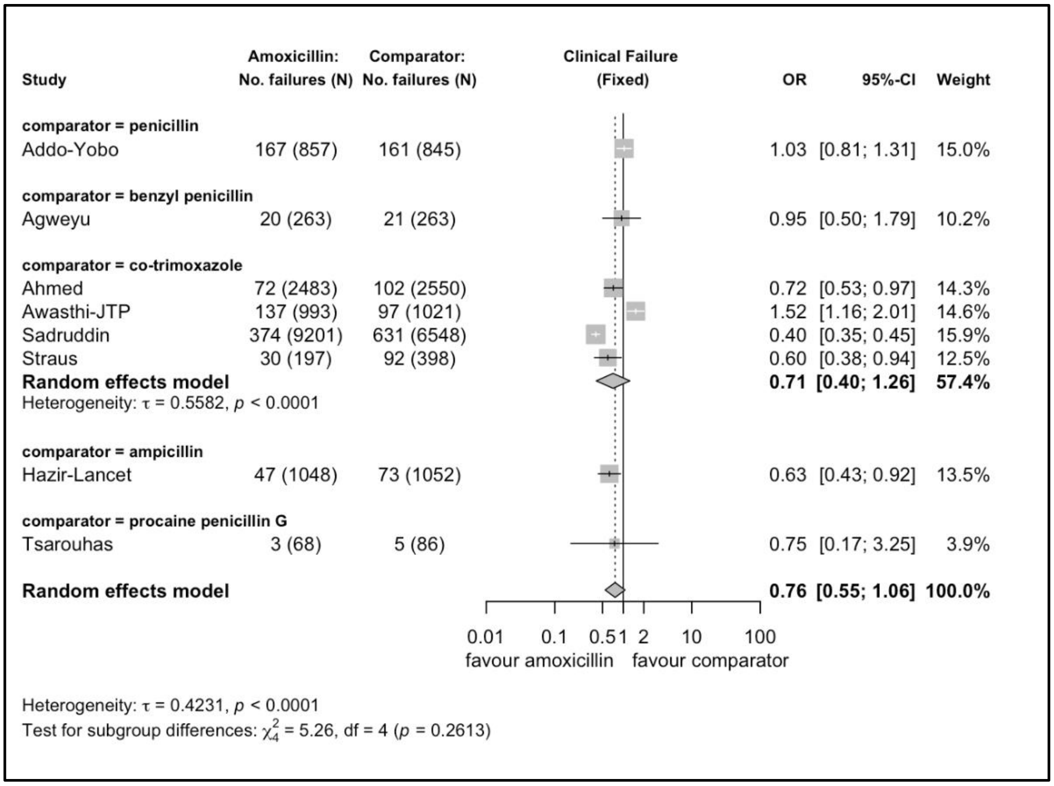

Supplementary Figure 5. Forest plot for fixed effects model of clinical failure (without placebo), amoxicillin only. One study (Tsarouhas_PEC) considers a patient population of greater than five years of age.

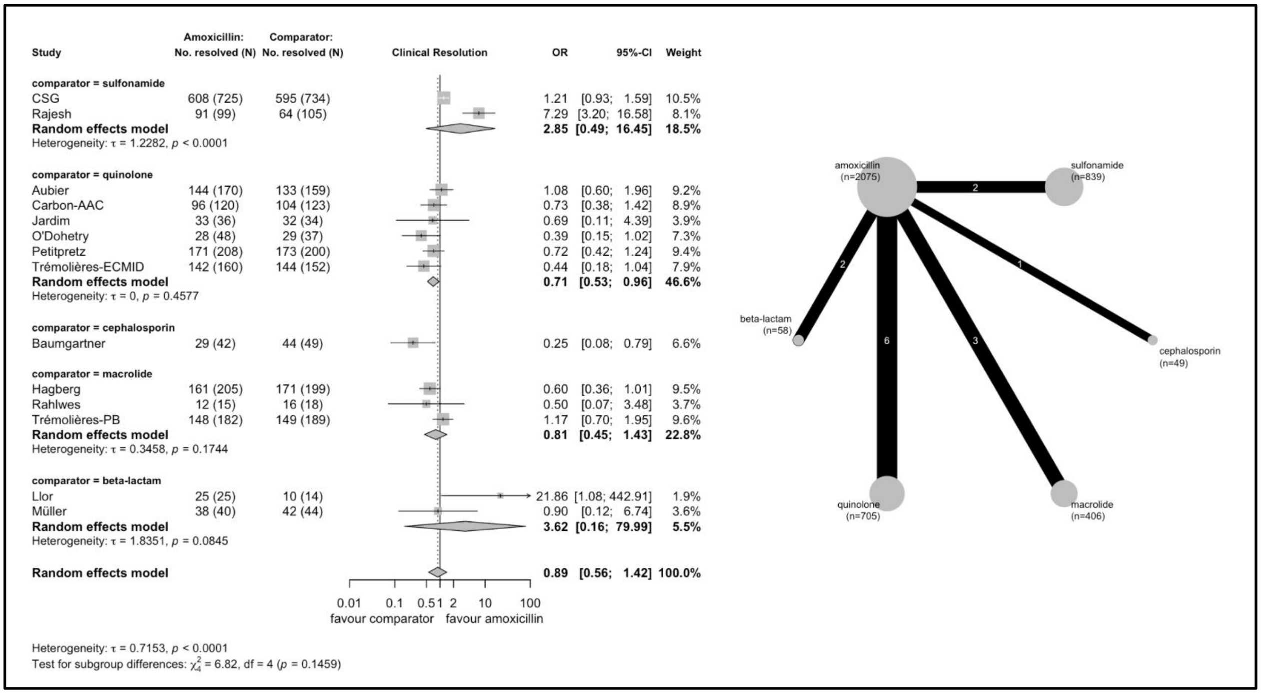

Supplementary Figure 6. Forest plot and network graph for clinical resolution by antibiotic class compared to amoxicillin.

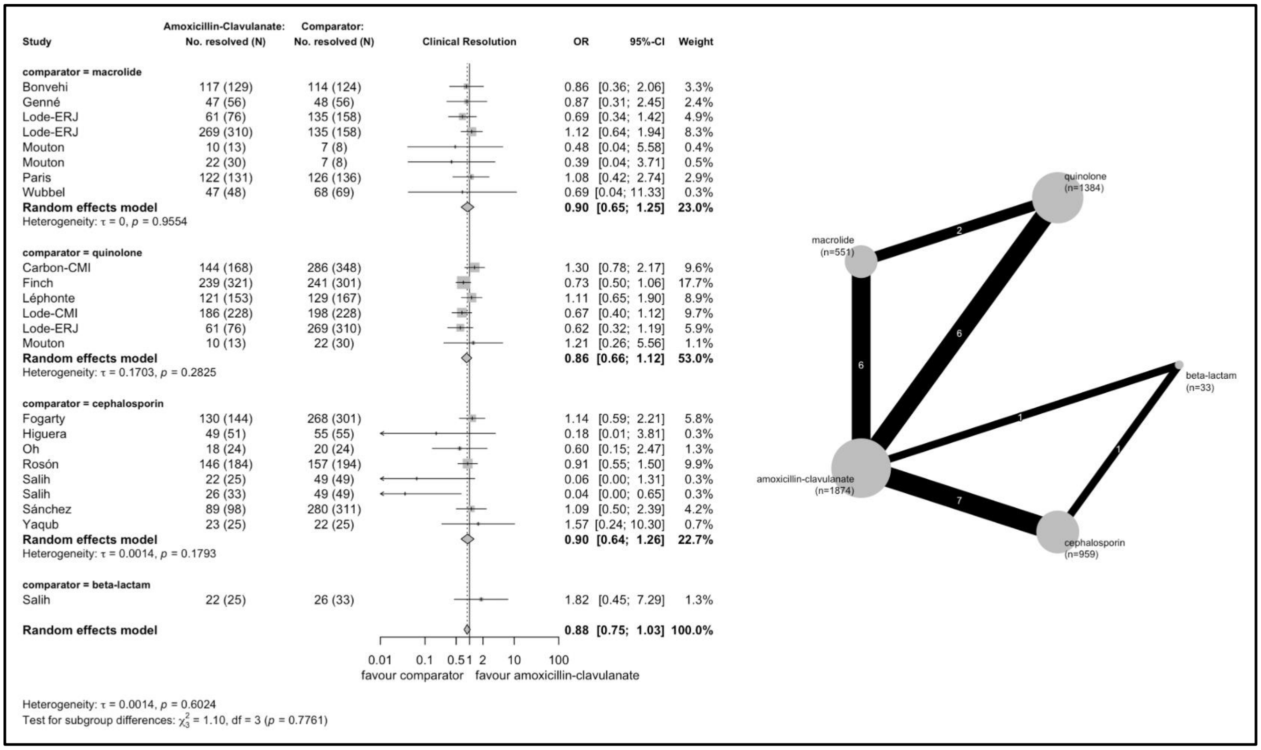

Supplementary Figure 7. Forest plot and network graph for clinical resolution by antibiotic class compared to amoxicillin-clavulanate.

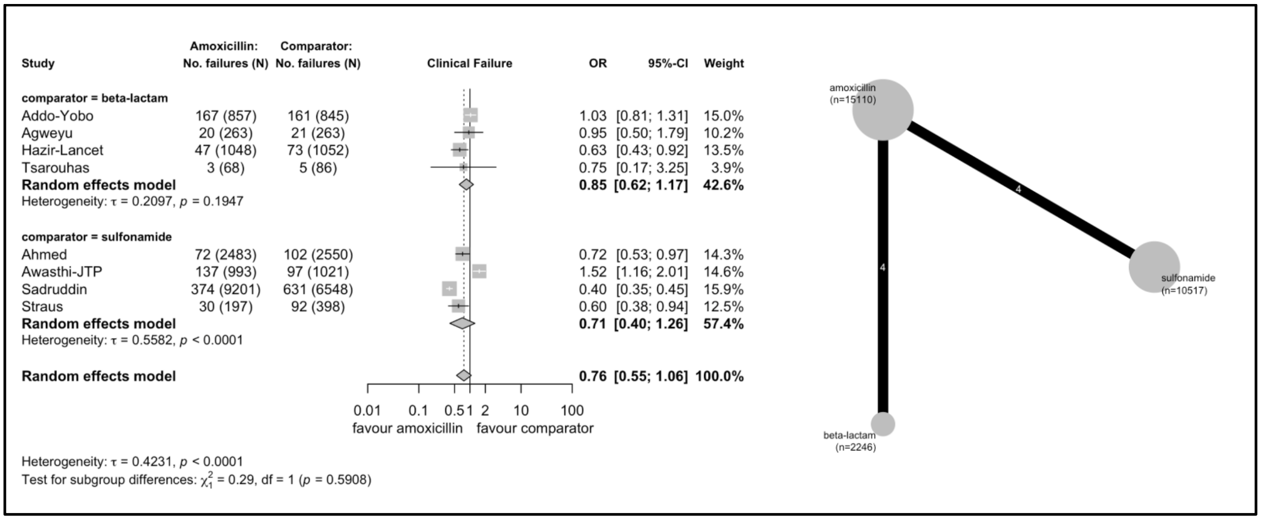

Supplementary Figure 8. Forest plot and network graph for clinical failure by antibiotic class (all amoxicillin comparator). One study (Tsarouhas) considered a patient population of greater than five years of age.

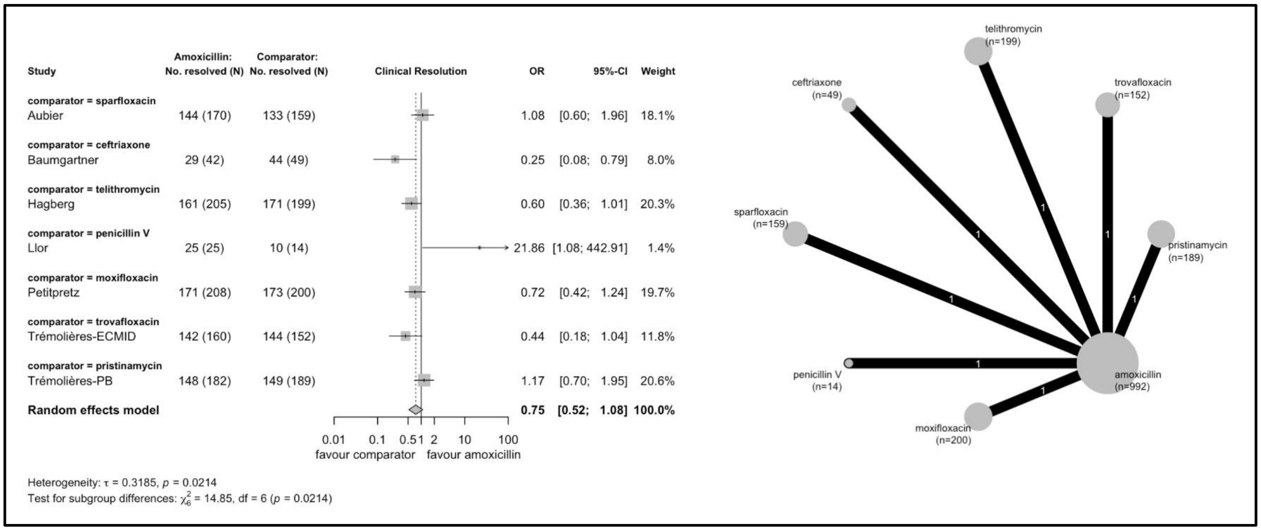

Supplementary Figure 9. Forest plot and network graph for clinical resolution and high dose of amoxicillin (≥80 mg/kg/day or 3000 mg/day). All patients were over five years of age.

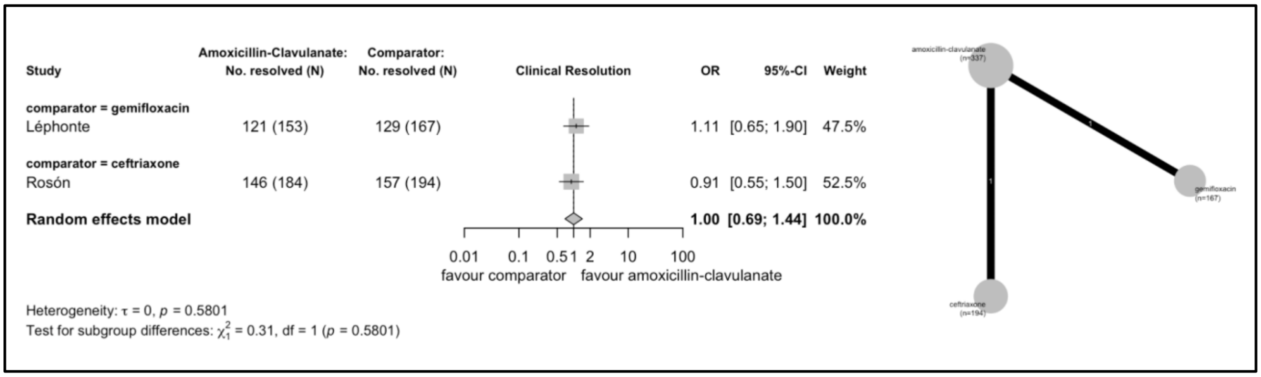

Supplementary Figure 10. Forest plot and network graph for clinical resolution and high dose of amoxicillin–clavulanate (≥80 mg/kg/day or 3000 mg/day). All patients were over five years of age.

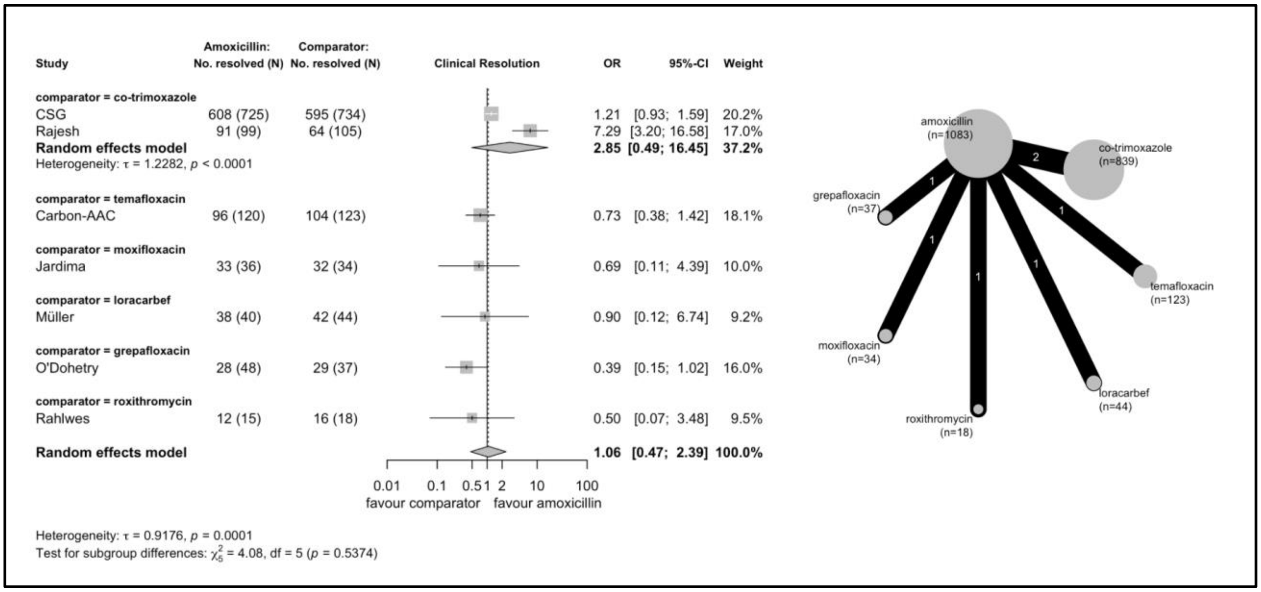

Supplementary Figure 11. Forest plot and network graph for clinical resolution and regular dose of amoxicillin. Two studies of patients less than five years of age (CSG, Rajesh).

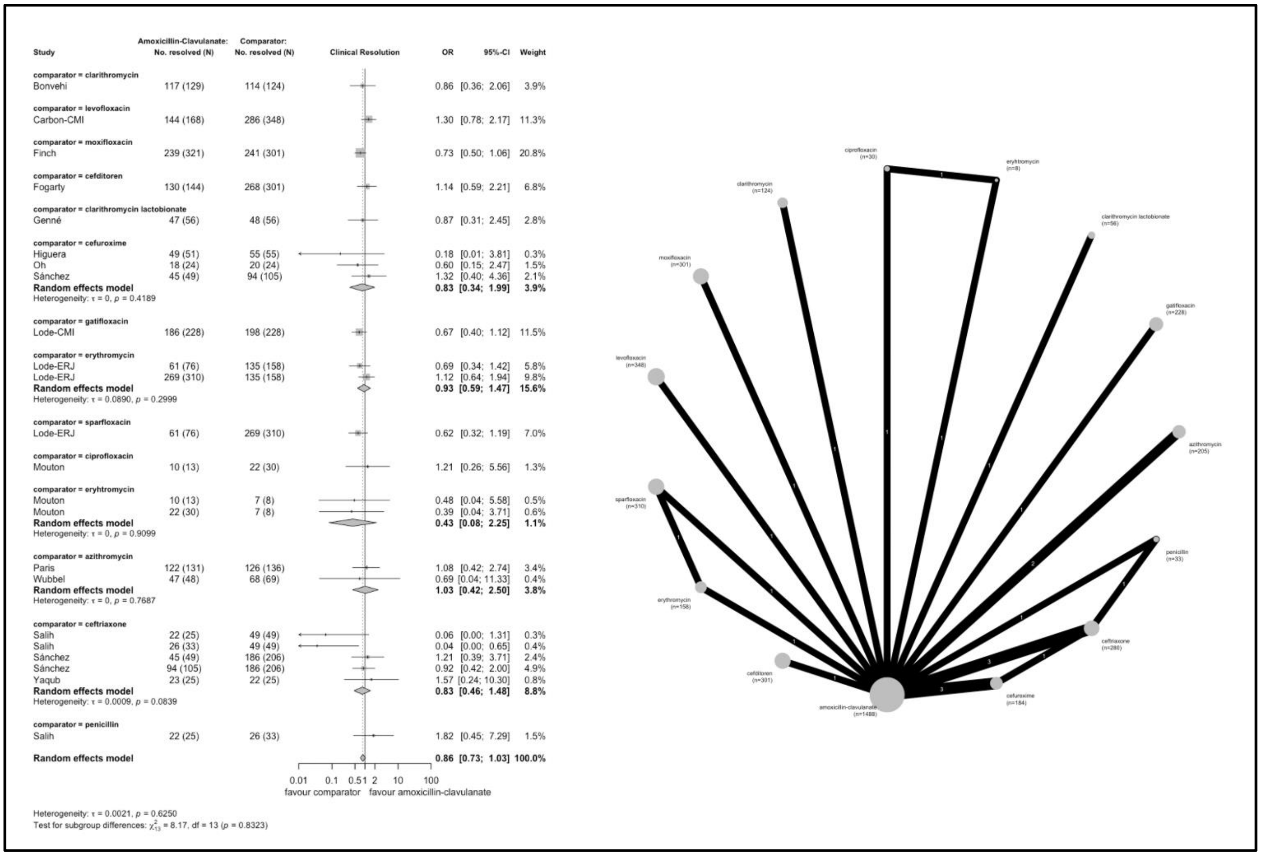

Supplementary Figure 12. Forest plot and network graph for clinical resolution and regular dose of amoxicillin-clavulanate. Two studies of patients less than five years of age (Salih, Wubbel).

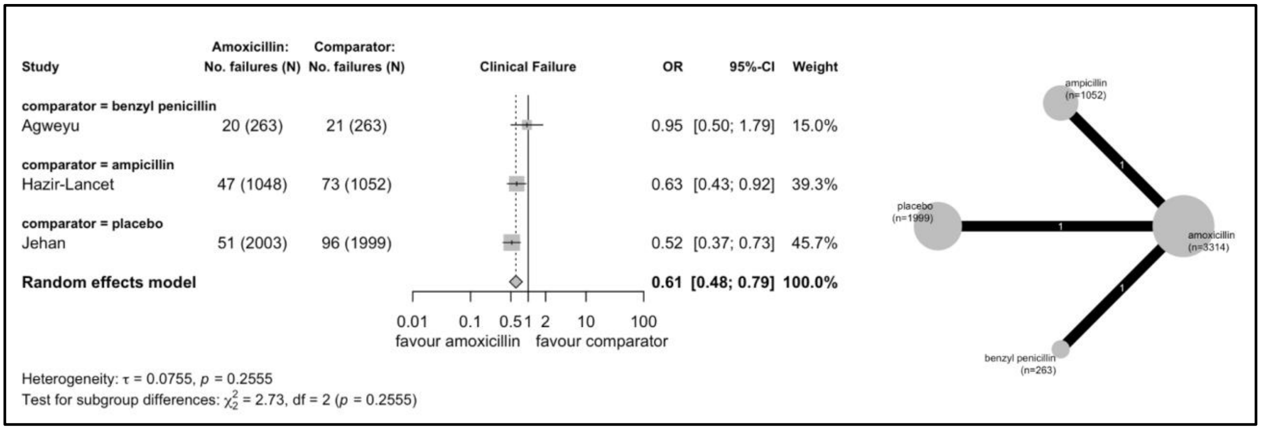

Supplementary Figure 13. Forest plot and network graph for clinical failure and high dose of amoxicillin. All studies considered patient populations less than five years of age.

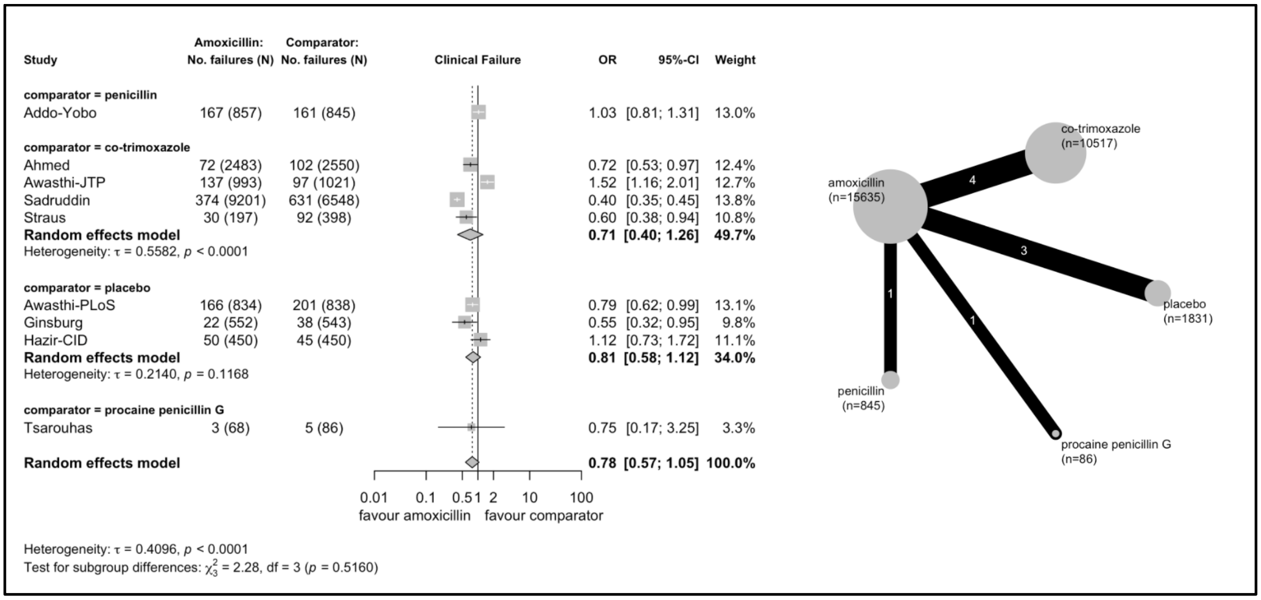

Supplementary Figure 14. Forest plot and network graph for clinical failure and regular dose of amoxicillin. One study (Tsarouhas) considered a patient population greater than five years of age.

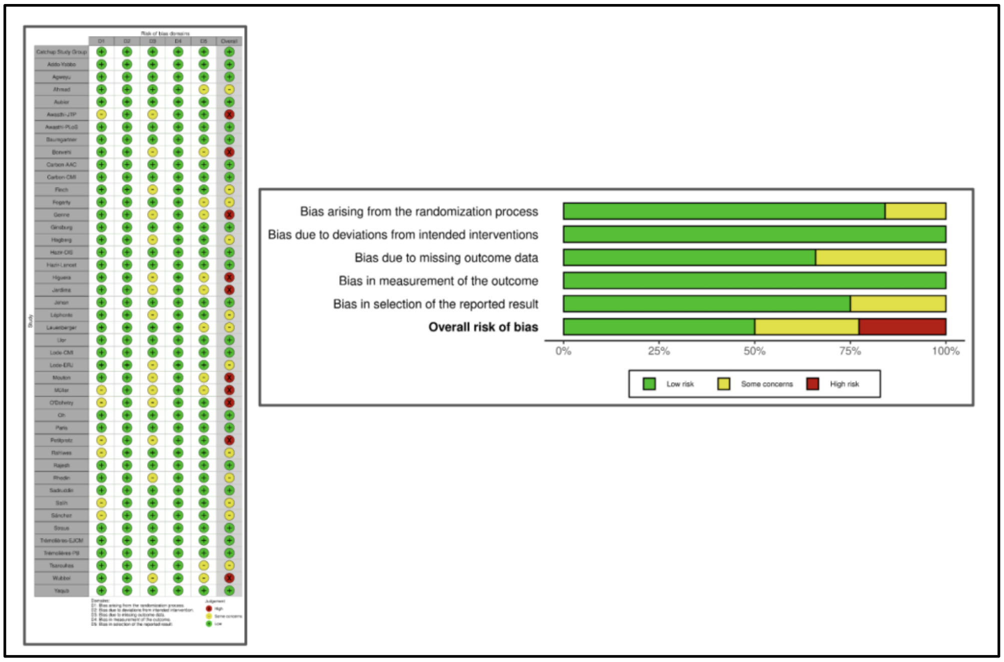

Supplementary Figure 15. Risk of bias assessments using the Cochrane RoB 2.0 assessment and R-based shiny web app risk of bias VISualization tool (robvis).

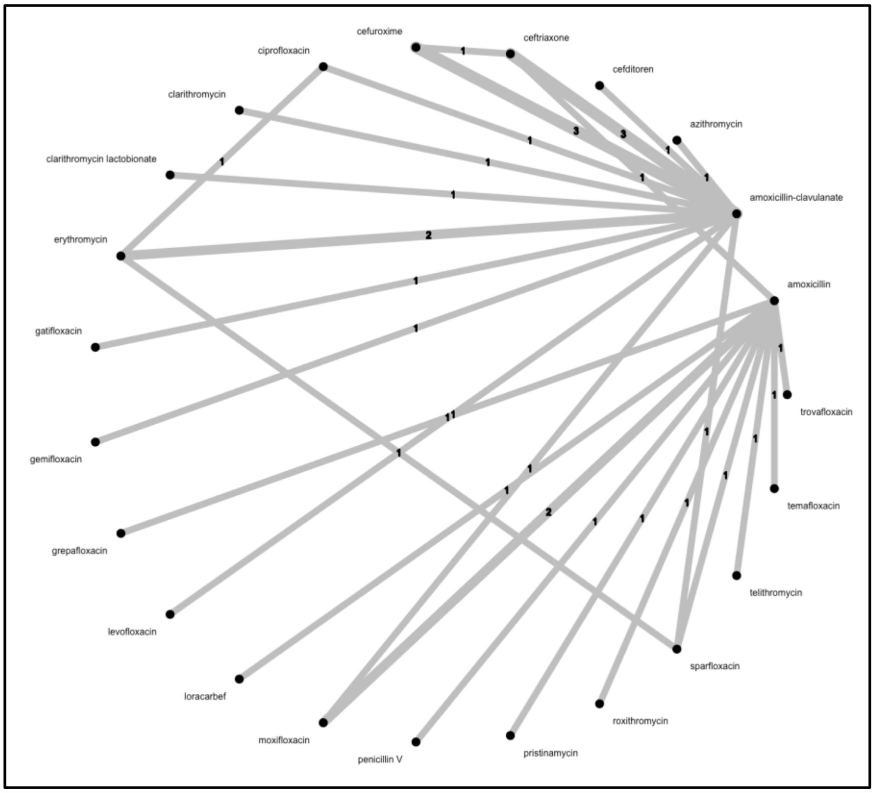

Supplementary Figure 16. Network wide meta-analysis of 28 studies considering adult CAP patients with a primary outcome of clinical resolution. Black edges indicate direct comparison between amoxicillin and a comparator antibiotic while grey edges indicate direct comparison

between amoxicillin-clavulanate and a comparator antibiotic. The numbers of studies comparing each antibiotic pair are detailed on the edge in the diagram.

Supplementary Table 2. Separating indirect from direct evidence (SIDE) using back-calculation method for the network meta-analysis.

Comparison	k	direct	indirect	RoR	z	p-value
ceftriaxone:amoxicillin	1	3.945	0.886	4.453	2.190	0.029
moxifloxacin:amoxicillin	2	1.392	1.483	0.938	-0.130	0.899
sparfloxacin:amoxicillin	1	0.924	2.229	0.415	-1.610	0.107
amoxicillin-clavulanate:ceftriaxone	3	0.982	0.248	3.956	2.380	0.018
amoxicillin-clavulanate:cefuroxime	3	0.826	0.698	1.182	0.220	0.824
amoxicillin-clavulanate:ciprofloxacin	1	1.212	2.878	0.421	-0.480	0.635
amoxicillin-clavulanate:erythromycin	2	0.672	2.811	0.239	-1.610	0.107
amoxicillin-clavulanate:moxifloxacin	1	0.726	0.773	0.938	-0.130	0.899
amoxicillin-clavulanate:sparfloxacin	1	0.620	1.357	0.457	-1.450	0.147
ceftriaxone:cefuroxime	1	1.088	0.772	1.410	0.440	0.662
ciprofloxacin:erythromycin	1	0.393	0.801	0.490	-0.480	0.635
erythromycin:sparfloxacin	1	0.895	4.993	0.179	-1.500	0.134

Comparison: Treatment comparison
k: Number of studies providing direct evidence
direct: Estimated treatment effect (OR) derived from direct evidence
indirect: Estimated treatment effect (OR) derived from indirect evidence
RoR: Ratio of Ratios (direct versus indirect)
z: z-value of test for disagreement (direct versus indirect)
p-value: p-value of test for disagreement (direct versus indirect)

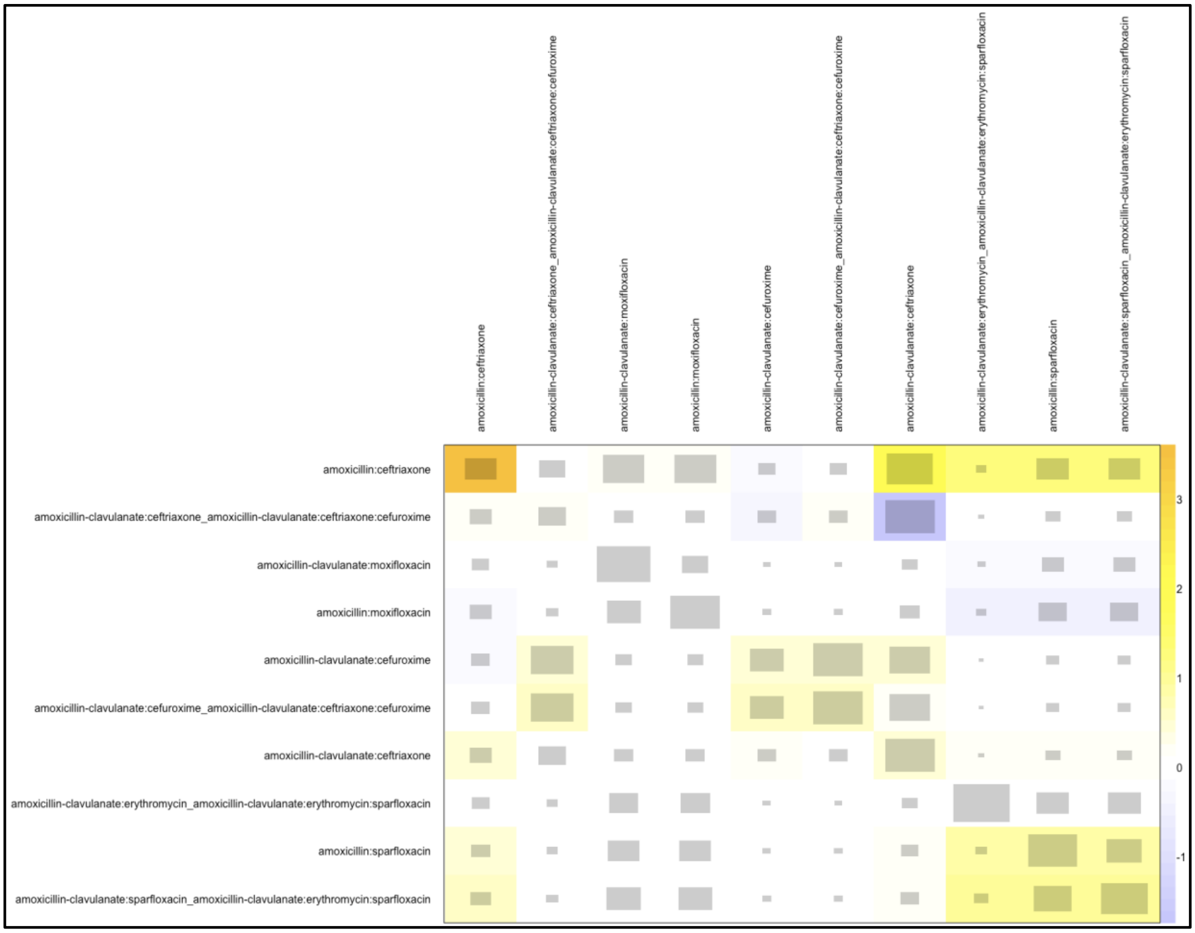

Supplementary Figure 17. Net heat plot of inconsistency between direct and indirect evidence for the network meta-analysis.

Supplementary Table 3. Distribution of age, setting, and severity among trials included in network meta-analysis.

**Study**	**Amoxicillin vs amoxicillin-clavulanate**	**Comparators**	**Age for amox arm**	**Age (spread for amox arm)**	**Study Setting**	**Amox arm Dosage**	**Severity rating[a] (1-4)**	**Approx. amox arm duration[b] (days)**
Aubier *et al* (1998)[16]	amoxicillin	sparfloxacin	Mean: 43.2 years	SD: 1.4 years	inpatient	high	1	10
Baumgartner *et al* (1984)[17]	amoxicillin	ceftriaxone	Mean: 60 years	SD: 19 years	inpatient	high	1	7
Bonvehi *et al* (2003)[29]	amoxicillin-clavulanate	clarithromycin	Mean: 46.7 years	SD: 18.5 years Range: 13-85 years	outpatient	regular	3	7
Carbon *et al* (1992)[18]	amoxicillin	temafloxacin	Mean: 55.6 years	SD: 19.1 years	inpatient	regular	1	10
Carbon *et al* (1999)[30]	amoxicillin-clavulanate	levofloxacin	Mean: 40.93 years	SD: 14.23 years	mixed	regular	2	8.5

Finch *et al* (2002)[31]	amoxicillin-clavulanate	moxifloxacin	Mean: 55.9 years	SD: 19.6 years	inpatient	regular	1	10.5
Fogarty *et al* (2002)[32]	amoxicillin-clavulanate	cefditoren	Mean: 50.0 years	Range: 13-93 years	outpatient	regular	3	14
Genné *et al* (1997)[33]	amoxicillin-clavulanate	clarithromycin	Mean: 69 years	SD: 16 years	inpatient	regular	1	10
Hagberg *et al* (2002)[19]	amoxicillin	telithromycin	Median: 43 years	Range: 18-88	mixed	high	2	10
Higuera *et al* (1996)[34]	amoxicillin-clavulanate	cefuroxime	Mean: 41.7 years	SEM: 2.5 Range: 12-89 years	outpatient	regular	3	10
Jardim *et al* (2003)[20]	amoxicillin	moxifloxacin	Mean: 48.6 years		mixed	regular	2	10
Léophonte *et al* (2004)*37*	amoxicillin-clavulanate	gemifloxacin	Mean: 55.3 years	SD: 19.8 years Range: 18-86 years	mixed	high	2	10
Leuenberger *et al* (1983)[22]	amoxicillin	cefaclor	Mean: 67.6 years	SEM: 4.3 years	inpatient	regular	1	7
Llor *et al* (2019)[23]	amoxicillin	penicillin	Mean: 49.0 years	SD: 14.3 years	outpatient	high	3	10
Lode *et al* (2004)[38]	amoxicillin-clavulanate	gatifloxacin	Mean: 50.2 years	SD: 17.28 years Range: 18-88 years	inpatient	regular	1	7.5
Lode *et al* (1995)[39]	amoxicillin-clavulanate	sparfloxacin, erythromycin	Mean: 52 years	SEM: 1 year	mixed	regular	2	9.5
Mouton *et al* (1991)[40]	amoxicillin-clavulanate	ciprofloxacin, erythromycin	Mean: 58.8 years	SD: 18.9 years	inpatient	regular	1	5
Müller *et al* (1992)[24]	amoxicillin	loracarbef	Mean: 50.6 years	SD: 18.2 years Range: 17.0-92.0 years	outpatient	regular	3	12
O'Dohertry *et al* (1997)[25]	amoxicillin	grepafloxacin	Mean: 55.0 years		outpatient	regular	3	8.5
Oh *et al* (1996)[35]	amoxicillin-clavulanate	cefuroxime	Mean: 39.25 years	SD: 17.2 years	inpatient	regular	1	7
Paris *et al* (2008)[41]	amoxicillin-clavulanate	azithromycin	Mean: 42.5 years	SD: 13.1 years Range: 14-76 years	outpatient	regular	3	7
Petitpretz *et al* (2001)[21]	amoxicillin	moxifloxacin	Median: 49.9 years	SD: 20.5 years	mixed	high	2	10
Rahlwes *et al* (1988)[26]	amoxicillin	roxithromycin	Mean age: 53.3 years		inpatient	regular	1	9.4
Rosón *et al* (2001)[43]	amoxicillin-clavulanate	ceftriaxone	Mean: 66 years		inpatient	high	1	10.9

Sánchez *et al* (1998)[36]	amoxicillin-clavulanate	cefuroxime, ceftriaxone	Mean: 61.7 years	SD: 17.4 years Range: 12-95 years	inpatient	regular	1	6
Trémolières *et al* (1998)[27]	amoxicillin	trovafloxacin	Mean: 52.2 years	Range: 16-92 years	mixed	high	2	8.5
Trémolières *et al* (2005)[28]	amoxicillin	pristinamycin	Mean: 47.2 years	SD: 17.7 years	inpatient	high	1	8.5
Yaqub *et al* (2005)[45]	amoxicillin-clavulanate	ceftriaxone	Mean: 54.1 years		inpatient	regular	1	7

[a] Severity score: We ranked the severity of community-acquired pneumonia (CAP) on a scale of one to four, with one indicating trials focused on non-severe cases and four indicating severe CAP cases. Due to inconsistencies in reporting, this enabled us to display the distribution of severity in reported cases. Trials
[b]Approximation duration of amoxicillin arm: Due to inconsistency in reporting, the approximate duration reflects either a reported measure of central tendency or a required duration for inclusion in the trial.
