# Supplementary material for: Effectiveness of amoxicillin and amoxicillin-clavulanate for the treatment of community-acquired pneumonia in adults and children: a systematic review and meta-analysis
Source: BMJ Open. 2026 May 7;16(5):e112219. doi: 10.1136/bmjopen-2025-112219 (PMC13157766; doi:10.1136/bmjopen-2025-112219)
Supplement: Supplementary file 2 [file bmjopen-2025-112219.reviewer_comments.pdf]

PEER REVIEW HISTORY

BMJ Open publishes all reviews undertaken for accepted manuscripts. Reviewers are asked to complete a checklist review form and are provided with free text boxes to elaborate on their assessment. These free text comments are reproduced below.

ARTICLE DETAILS

Title (Provisional)

Effectiveness of amoxicillin and amoxicillin-clavulanate for the treatment of community-acquired pneumonia in adults and children: a systematic review and meta-analysis

Authors

Potter-Schwartz, Lilia; Swe, Myo MM; Sharland, Mike; Bielicki , Julia Anna; Cooper, Ben S; Lim, Cherry

---

VERSION 1 - REVIEW

**Reviewer**	**1**
**Name**	**Noma, Hisashi**
**Affiliation**	**The Institute of Statistical Mathematics, Department of Interdisciplinary Statistical Mathematics**
**Date**	**02-Nov-2025**
**COI**	**None**

This manuscript presents a systematic review and meta-analysis comparing the effectiveness of amoxicillin (AMX) and amoxicillin-clavulanate (AMC) for the treatment of community-acquired pneumonia (CAP).

1. Although some closed loops may exist within the network, the authors did not report any quantitative assessment of inconsistency (e.g., loop-specific inconsistency factors, node-splitting, or design-by-treatment interaction models). These analyses are essential and must be conducted.

2. Many comparisons do not form closed loops and therefore depend heavily on the transitivity assumption. However, a Transitivity Table as recommended by Salanti et al. (2014, https://doi.org/10.1371/journal.pone.0099682)—showing the distribution of key effect modifiers such as age, disease severity, region, diagnostic criteria, dosage, and follow-up duration—was not provided, and there is no discussion or sensitivity analysis regarding the plausibility of this assumption. A Transitivity Table should at least be added to the Supplement, accompanied by an explicit examination of the plausibility of transitivity and, if needed, relevant sensitivity analyses.

3. The assessment of heterogeneity is limited to a single statement that it was evaluated using the I^2 statistic, but even the I^2 values are not reported. No information is provided on τ^2 (between-study variance), the Q statistic, or prediction intervals.

4. The analytical model is described as a “random effects logistic regression,” but it is unclear whether it includes only a study-specific random intercept (a simple GLMM) or also random slopes. Details regarding the estimation of τ^2 and the specific methods used for heterogeneity assessment are missing.

5. Logistic mixed-effects or logistic regression models can only yield odds ratios. As Altman, Deeks and Sackett (1998, https://doi.org/10.1136/bmj.317.7168.1318) noted, odds ratios are difficult to interpret except as approximations to risk ratios under rare-event conditions. The authors should justify why logistic regression is an appropriate and meaningful choice of model for this research.

6. Given that some analyses include only a limited number of studies, it would be preferable to apply the Hartung–Knapp–Sidik–Jonkman (HKSJ) method, as recommended by the Cochrane Handbook, for more reliable inference.

7. Several mandatory items required by PRISMA-NMA are missing: inconsistency assessment (methods and results), evaluation of transitivity (assumptions, effect modifiers, sensitivity analysis), reporting of τ^2, I^2, and Q statistics, and clear differentiation between direct and indirect evidence for each comparison. The authors should review the PRISMA-NMA 25-item checklist and supplement all missing elements.

8. The integration of pediatric and adult studies into a single meta-analysis raises concerns about heterogeneity due to effect modification.

9. The authors state, “We simulated 50 studies to demonstrate the utility of network meta-analysis,” but the purpose, design, and role of this simulation are highly unclear. It should be explicitly stated whether this was intended as a methodological illustration or as part of the main analysis. Its relevance to the study’s clinical question is questionable, and as a methodological contribution, the level of rigor is low. If the authors believe this simulation exercise has independent value, it should be developed into a separate methodological paper rather than included here.

10. The layout and organization of figures and tables are confusing and impede comprehension of the results section.

**Reviewer**	**2**
**Name**	**Van Praet, Jens**
**Affiliation**	**AZ Sint-Jan Brugge-Oostende AV**
**Date**	**20-Dec-2025**
**COI**	**None**

This is a well conducted study and a well written manuscript.

Minor comment:

I could not open the links to github.

**Reviewer**	**3**
**Name**	**Treadwell, Jonathan**
**Affiliation**	**ECRI, Technology Assessment Group**
**Date**	**23-Dec-2025**
**COI**	**None**

BMJ review Dec 2025

Effectiveness of amoxicillin and amoxicillin-clavulanate for the treatment of community-acquired pneumonia in adults and children: systematic review and meta-analysis

Jonathan R Treadwell, ECRI, Willow Grove PA, USA

Major comments

Your abstract states "Our study found no strong evidence, from the published trials, of difference in clinical outcomes of amoxicillin compared to amoxicillin-clavulanate or other antibiotics for CAP treatment. However, there are large uncertainties around the pooled estimates". My problem with this wording is that the clinical question should not be whether there is "strong" evidence. It should be whether you drew any conclusion about whether there is an effect. And apparently the confidence intervals are too wide for that. So I think a better overall summary is something like "Evidence is unclear about whether amoxicillin or amoxicillin-clavulanate differ from other antibiotics in effectiveness at treating community-acquired pneumonia." You also use the wording "no evidence of difference", but the problem with that is that the reader doesn't know whether you are 1) concluding that there IS no difference, or 2) that you are simply not drawing a conclusion. It appears that you are in the latter camp. Your results are also inconclusive for amoxicillin vs amoxicillin-clavulanate. Text in the discussion also suffers from these same issues.

I was surprised that there was no mention of the similarity and consistency assumptions of NMA or the testing of such assumptions. See https://onlinelibrary.wiley.com/doi/full/10.1002/jrsm.1760

Interesting that NONE of the 44 studies reported BOTH resolution and failure. Could your discussion section theorize as to the reason(s)? I presume one is not the converse of the other, or else you could've done computations to include them both in the same analysis. Surely the two outcomes are related (higher resolution means lower failure) but I understand they really are different outcomes. There is the third possibility of neither resolution nor failure. Perhaps you could do an analysis whereby you assume X% of patients were in this neutral category and

you then combine your two primary outcomes. Eg we assume X=20%...if one study reported 70% resolution, and then we suppose 10% failure. Or if another study had 20% failure then we suppose 60% resolution. And we then can have data on resolution even in studies that only reported failure (via the assumption about X). Then you vary X and see if any specific level of X points to any clear conclusions from the network. Just as a hypothetical….the large CIs would motivate me to try to combine the two primary outcomes, as they are two sides of (what I suspect) is a three-sided coin? But perhaps I am revealing my ignorance in this area.

Minor comments

Very well-written

“Lower respiratory infections were the fourth highest contributor to global disability-adjusted life years (DALYs) in 2019.” Can you find any more recent years than 6 years ago? Or maybe this is an intentional date because it’s pre-pandemic?

The last search was 17 months ago. I understand it’s a lot of work to update the search, but readers might wonder if any additional trials have been published since then. Consider a search that you just discuss any more recent trials, rather than the painstaking process of updating all your meta-analyses.

“Additionally, studies were excluded if patients with other infections besides CAP were randomized, multiple antibiotics were used in the amoxicillin arm (i.e. combined treatment of amoxicillin with other antibiotics), or the primary outcome was neither clinical resolution nor clinical failure”. Technically this means that if only a single patient in a trial had received additional antibiotics, or if the trial had reported clinical resolution/failure as a secondary outcome, then you would exclude the trial. These seem overly stringent. Perhaps you didn’t mean those things, in which case, consider rewording.

Agree with the review authors choice of primary and secondary outcomes. But in terms of study inclusion, it shouldn’t really matter what the study authors defined as their primary and secondary outcomes. It might matter in terms of the risk of bias domain selection of the reported results.

Great they shared data and code…but I went to https://github.com/liliapsnh/cap_amoxicillin.git and got an error message. Same for https://github.com/Cherrylim128/AmoxForCap

Tau is a more direct measure of heterogeneity than I2. See the article by Rucker in BMC Med Res Methodol 8(1) p79. The problem with I2 is that it depends on the Ns. Try it yourself: take a meta-analysis and look at I2. Now multiply all the Ns by 3, but keep all the effect sizes the same. See how I2 increased? Yeah. That’s not good. Note that tau would not have increased, since it more purely measures the extent to which effect sizes differed.

Curious about the risk of bias domain “selection of reported result” and on what basis some studies were penalized. Did they not report all the outcomes that their protocols said they’d collect? Or maybe their methods section mentioned more outcomes than their results sections provided? Did this have anything to do with them reporting clinical resolution or clinical failure?

I'm puzzled about the "Conclusion" sentence "Our findings provide a strong rationale for the WHO AWaRe book recommendation of using amoxicillin as a first-choice antibiotic for the treatment of CAP in adults and children". The main purpose of this work is not to investigate the effectiveness of amoxicillin itself, but rather to compare it to other antibiotics. So it looks odd to make that to focus of the overall "Conclusion".

Regarding fixed effects. Figure 5 is the one comparing amoxicillin amoxicillin to placebo and it used a fixed effects model even though I^2 was 67%. I would think random effects would be more appropriate, given that heterogeneity. The methods section said "We also evaluated the effect of amoxicillin against other antibiotic treatment on outcomes using fixed-effects logistic regression models as a sensitivity analyses, assuming there was a true underlying effect of amoxicillin on the outcome across the studies included in the meta-analysis." However the justification for fixed effects should be not that we assume there IS an effect, but that all studies are measuring the same underlying MAGNITUDE of effect, which is a rarely made assumption.

VERSION 1 - AUTHOR RESPONSE

Reviewer: 1

Prof. Hisashi Noma, The Institute of Statistical Mathematics, The Graduate University for Advanced Studies

- Although some closed loops may exist within the network, the authors did not report any quantitative assessment of inconsistency (e.g., loop-specific inconsistency factors, node-splitting, or design-by-treatment interaction models). These analyses are essential and must be conducted.

  Thank you for the helpful comment. We have performed and reported quantitative assessment of inconsistency and added the text below in the "Methods" section:

  "Finally, we conducted an unplanned frequentist random-effects network meta-analysis to compare amoxicillin and amoxicillin–clavulanate, assuming that each study had its own true effect. Network meta-analysis relies on the transitivity assumption—that the distribution of effect modifiers is similar across treatment comparisons. To mitigate the risk of violating this assumption, we restricted the analysis to trials involving adult patients only. Furthermore, we assessed the transitivity assumption by examining the distribution of potential effect modifiers—age, setting, and dosage—across treatment comparisons. In addition, net heat plots and the node-splitting method were used to evaluate agreement between direct and indirect evidence in the network (i.e., the consistency assumption). The netmeta package was used for the network plots and network meta-analysis."

  The text below has been added in the results section to report results from the analyses:

  "The global Q score for inconsistency was 7.36 (p-value=0.195), and the only comparisons that were inconsistent were ceftriaxone versus amoxicillin (p-value 0.021) and ceftriaxone versus amoxicillin-clavulanate (p-value 0.0175) (Supplementary Table 2, Supplementary Figure 17). However, the tests have limited power as the network of the 28 studies was sparse. We found

no clear evidence of violations of the transitivity assumption when comparing age, setting, and dosage, of studies across the comparisons (Supplementary Table 3). However, the number of studies per comparison was small."

- Many comparisons do not form closed loops and therefore depend heavily on the transitivity assumption. However, a Transitivity Table as recommended by Salanti et al. (2014, https://doi.org/10.1371/journal.pone.0099682)—showing the distribution of key effect modifiers such as age, disease severity, region, diagnostic criteria, dosage, and follow-up duration—was not provided, and there is no discussion or sensitivity analysis regarding the plausibility of this assumption. A Transitivity Table should at least be added to the Supplement, accompanied by an explicit examination of the plausibility of transitivity and, if needed, relevant sensitivity analyses.

  Thank you for the suggestion and sharing the reference. We have included a table to show the distribution of potential effect modifiers including age, setting, and dosage. This information has been added to our supplemental material (Supplementary Table 3), as well as the methods and results sections of our manuscript.

- The assessment of heterogeneity is limited to a single statement that it was evaluated using the I^2 statistic, but even the I^2 values are not reported. No information is provided on τ^2 (between-study variance), the Q statistic, or prediction intervals.

  Thank you for the comment. The text below has been added to report the global Q score for inconsistency and local consistency:
  "The global Q score for inconsistency was 7.36 (p-value=0.195), and the only comparisons that were inconsistent were ceftriaxone versus amoxicillin (p-value 0.021) and ceftriaxone versus amoxicillin-clavulanate (p-value 0.0175) (Supplementary Table 2, Supplementary Figure 17)."

  The detailed analysis outputs on the assessment of local inconsistency has been reported in Supplementary Table 2.

- The analytical model is described as a "random effects logistic regression," but it is unclear whether it includes only a study-specific random intercept (a simple GLMM) or also random slopes. Details regarding the estimation of τ^2 and the specific methods used for heterogeneity assessment are missing.

  The text below was added to clarify the assumption of the random effect model:
  "We evaluated the effect of amoxicillin against alternative antibiotics on outcomes using a random effects logistic regression model, assuming that each study had its own magnitude of effect, to include between-study variability into the estimation of the overall treatment effects."

- Logistic mixed-effects or logistic regression models can only yield odds ratios. As Altman, Deeks and Sackett (1998, https://doi.org/10.1136/bmj.317.7168.1318) noted, odds ratios are difficult to interpret except as approximations to risk ratios under rare-event conditions. The authors should justify why logistic regression is an appropriate and meaningful choice of model for this research.

We thank the reviewer for raising this point. We agree that odds ratios can sometimes be difficult to interpret when event rates are high. In our study, however, the outcome of clinical failure was rare (median: 6.5%), such that the odds ratio closely approximates the risk ratio. We followed the pre-specified analysis plan which was developed on an assumption of a rare outcome and therefore used odds ratios. Moreover, the fixed-effect (assuming there is an overarching magnitude of effect) and random-effects (assuming each trial has its own magnitude of effect) logistic regression models performed in the planned meta-analysis did not include any covariates, therefore non-collapsibility is not a concern in the interpretation of the our findings.

- Given that some analyses include only a limited number of studies, it would be preferable to apply the Hartung–Knapp–Sidik–Jonkman (HKSJ) method, as recommended by the Cochrane Handbook, for more reliable inference.

  Thank you for your comment. We conducted additional sensitivity analyses separately for the two main meta-analyses of amoxicillin and amoxicillin–clavulanate using the Hartung–Knapp–Sidik–Jonkman method. The text describing these analyses has been added to the "Methods" section as below:

  "An additional sensitivity analysis was performed using the Hartung–Knapp–Sidik–Jonkman method."

  The text below to report the findings from the sensitivity analyses has been added in the "Results" section:

  "The findings from the sensitivity analysis using the Hartung–Knapp–Sidik–Jonkman method showed similar patterns with wider uncertainty intervals (amoxicillin treatment compared to other treatment: 0.88; 95% CI: 0.53-1.47; τ=0.70; amoxicillin-clavulanate treatment compared to other treatment: 0.89; 95% CI: 0.76-1.03; τ=0)."

  "The sensitivity analysis using the Hartung–Knapp–Sidik–Jonkman method gave similar findings with wider uncertainty intervals (amoxicillin treatment compared to other treatment: 0.76; 95% CI: 0.55-1.06; τ=0.42)."

  We note that the Cochrane handbook states that "The (HKSJ) adjustment widens the confidence interval to reflect uncertainty in the estimation of between-study heterogeneity, and performs well in the presence of observed heterogeneity, except when there are few studies, when it can yield overly wide confidence intervals (Röver et al 2015)". We therefore have opted to use this method only for this sensitivity analysis.

- Several mandatory items required by PRISMA-NMA are missing: inconsistency assessment (methods and results), evaluation of transitivity (assumptions, effect modifiers, sensitivity analysis), reporting of τ^2, I^2, and Q statistics, and clear differentiation between direct and indirect evidence for each comparison. The authors should review the PRISMA-NMA 25-item checklist and supplement all missing elements.

Thank you for your comment. We have added the completed PRISMA-NMA guidelines to our file submission, as well as the necessary components to our manuscript and analysis.

- The integration of pediatric and adult studies into a single meta-analysis raises concerns about heterogeneity due to effect modification.

  Given that our systematic review and meta-analysis sought to evaluate existing evidence on the effectiveness of amoxicillin and amoxicillin-clavulanate for CAP for children and adults, we took specific steps to ensure that subgroup analyses would provide appropriate analyses. As mentioned previously, in our PROSPERO protocol, we identified prespecified subgroup analysis to include age-group (<5 years, >5 years of age). For each subgroup analysis, we have calculated and interpreted the respective level of heterogeneity.

- The authors state, "We simulated 50 studies to demonstrate the utility of network meta-analysis," but the purpose, design, and role of this simulation are highly unclear. It should be explicitly stated whether this was intended as a methodological illustration or as part of the main analysis. Its relevance to the study's clinical question is questionable, and as a methodological contribution, the level of rigor is low. If the authors believe this simulation exercise has independent value, it should be developed into a separate methodological paper rather than included here.

  We have removed this section from the manuscript.

- The layout and organization of figures and tables are confusing and impede comprehension of the results section.

  Thank you for your feedback on the layout and organization. Given that the network meta-analysis was ad-hoc, we have now reordered the supplementary figures to emphasize the pairwise meta-analysis. A key finding from our systematic review was that no trial directly compared amoxicillin and amoxicillin-clavulanate. Hence, we performed an unplanned network meta-analysis to explore the indirect evidence for difference in effectiveness between the two treatments.

Reviewer: 2
Dr. Jens Van Praet, AZ Sint-Jan Brugge-Oostende AV, Ghent University
Comments to the Authors:
This is a well conducted study and a well written manuscript.
Minor comment:

- I could not open the links to github.
  Our apologies about the error with the github link. We have updated access.

Reviewer: 3
Dr. Jonathan Treadwell, ECRI
Comments to the Authors:

- Your abstract states “Our study found no strong evidence, from the published trials, of difference in clinical outcomes of amoxicillin compared to amoxicillin-clavulanate or other antibiotics for CAP treatment. However, there are large uncertainties around the pooled estimates”. My problem with this wording is that the clinical question should not be whether there is “strong” evidence. It should be whether you drew any conclusion about whether there is an effect. And apparently the confidence intervals are too wide for that. So I think a better overall summary is something like “Evidence is unclear about whether amoxicillin or amoxicillin-clavulanate differ from other antibiotics in effectiveness at treating community-acquired pneumonia.” You also use the wording “no evidence of difference”, but the problem with that is that the reader doesn’t know whether you are 1) concluding that there IS no difference, or 2) that you are simply not drawing a conclusion. It appears that you are in the latter camp. Your results are also inconclusive for amoxicillin vs amoxicillin-clavulanate. Text in the discussion also suffers from these same issues.

  Thank you for the suggestion and note about this. We have updated the conclusion in the abstract as below:
  “Current evidence is unclear as to whether amoxicillin or amoxicillin–clavulanate differ from other antibiotics, or from each other, in the treatment of community-acquired pneumonia, owing to the small number of trials and substantial heterogeneity in comparators used across study settings.”

- I was surprised that there was no mention of the similarity and consistency assumptions of NMA or the testing of such assumptions. See https://onlinelibrary.wiley.com/doi/full/10.1002/jrsm.1760

  Thank you for pointing out the missing statistical analysis. We have performed the statistical test for the consistency assumption and added the description in the “Methods” section as below:

  “Finally, we conducted an unplanned frequentist random-effects network meta-analysis to compare amoxicillin and amoxicillin–clavulanate, assuming that each study had its own true effect. Network meta-analysis relies on the transitivity assumption—that the distribution of effect modifiers is similar across treatment comparisons. To mitigate the risk of violating this assumption, we restricted the analysis to trials involving adult patients only. Furthermore, we assessed the transitivity assumption by examining the distribution of potential effect modifiers—age, setting, and dosage—across treatment comparisons. In addition, net heat plots and the node-splitting method were used to evaluate agreement between direct and indirect evidence in the network (i.e., the consistency assumption). The netmeta package was used for the network plots and network meta-analysis.”

  The results have been reported in the “Results” section as below:

  “The global Q score for inconsistency was 7.92 (p-value=0.161), and the only comparison that was inconsistent was ceftriaxone versus amoxicillin (p-value = 0.021) (Supplementary Table 2, Supplementary Figure 17). However, the tests have limited power as the network of the 28 studies was sparse. We found no clear evidence of violations of the transitivity assumption

when comparing age, setting, and dosage, of studies across the comparisons (Supplementary Table 3). However, the number of studies per comparison was small."

- Interesting that NONE of the 44 studies reported BOTH resolution and failure. Could your discussion section theorize as to the reason(s)? I presume one is not the converse of the other, or else you could've done computations to include them both in the same analysis. Surely the two outcomes are related (higher resolution means lower failure) but I understand they really are different outcomes. There is the third possibility of neither resolution nor failure. Perhaps you could do an analysis whereby you assume X% of patients were in this neutral category and you then combine your two primary outcomes. Eg we assume X=20%...if one study reported 70% resolution, and then we suppose 10% failure. Or if another study had 20% failure then we suppose 60% resolution. And we then can have data on resolution even in studies that only reported failure (via the assumption about X). Then you vary X and see if any specific level of X points to any clear conclusions from the network. Just as a hypothetical….the large CIs would motivate me to try to combine the two primary outcomes, as they are two sides of (what I suspect) is a three-sided coin? But perhaps I am revealing my ignorance in this area.

  Thank you for your comment on this. We have added an additional sensitivity analysis to demonstrate the similarity in results when combining these two primary outcomes. For this analysis, we considered patients in the "clinical failure" trial who did not "fail" treatment as having been a case of "clinical resolution," excluding patients who were lost to follow-up. This added sensitivity analysis, which combined the eight clinical failure studies and the 15 clinical resolution studies comparing amoxicillin to an alternative antibiotic treatment, yielded an odds ratio of 1.06 (95% CI: 0.79, 1.42) where >1 favours amoxicillin (Supplementary Figure 15). In comparison, the clinical-failure subset of these studies yielded an odds ratio of 0.76 (95% CI: 0.55, 1.06) where <1 favours amoxicillin (Figure 4) and the clinical-resolution subset of these studies yielded an odds ratio of 0.88 (95% CI: 0.56, 1.38) where >1 favours amoxicillin (Figure 2).

  We have described this analysis in our methods section:
  "We also conducted a sensitivity analysis where we combined the "clinical failure" and "clinical resolution" studies comparing amoxicillin to an alternative antibiotic treatment by considering patients in the "clinical failure" trials who did not reach the clinical failure endpoint to have achieved clinical resolution, excluding patients lost to follow-up."

  And we added in our results section:
  "The sensitivity analysis that combined the eight clinical failure and 15 clinical resolution studies comparing amoxicillin to an alternative antibiotic treatment yielded an odds ratio of 1.06 (95% CI: 0.79, 1.42) were >1 favours amoxicillin for clinical resolution (Supplementary Figure 15).

- "Lower respiratory infections were the fourth highest contributor to global disability-adjusted life years (DALYs) in 2019." Can you find any more recent years than 6 years ago? Or maybe this is an intentional date because it's pre-pandemic?

  We have updated this statistic to reflect research published in December, 2025 in *The Lancet Infectious Diseases* as part of the Global Burden of Diseases, Injuries, and Risk Factors Study

(GBD) 2023. The analysis found that lower respiratory infections remain the world's leading infectious cause of death.

- The last search was 17 months ago. I understand it's a lot of work to update the search, but readers might wonder if any additional trials have been published since then. Consider a search that you just discuss any more recent trials, rather than the painstaking process of updating all your meta-analyses.

  We have conducted a search to identify any articles published between our last search date (July 16, 2024) and January 20, 2026. We followed the same search protocol outlined in the main text.

  This new search has yielded one additional study, published in the Chinese Journal of Infection and Chemotherapy in 2025. In this study, the authors found that amoxicillin-clavulanate IV infusion for 7-14 days was noninferior to ampicillin-sulbactam in terms of clinical resolution and microbiological efficacy in the treatment of CAP among adult patients. These results align with our findings of an unclear difference between amoxicillin or amoxicillin-clavulanate and comparator antibiotics. We have updated the discussion to include this research and our efforts to identify more recent trials.

- "Additionally, studies were excluded if patients with other infections besides CAP were randomized, multiple antibiotics were used in the amoxicillin arm (i.e. combined treatment of amoxicillin with other antibiotics), or the primary outcome was neither clinical resolution nor clinical failure". Technically this means that if only a single patient in a trial had received additional antibiotics, or if the trial had reported clinical resolution/failure as a secondary outcome, then you would exclude the trial. These seem overly stringent. Perhaps you didn't mean those things, in which case, consider rewording.

  We have reworded this statement to ensure the exclusion of studies is more clear.

- Agree with the review author's choice of primary and secondary outcomes. But in terms of study inclusion, it shouldn't really matter what the study authors defined as their primary and secondary outcomes. It might matter in terms of the risk of bias domain selection of the reported results.

  Thank you for highlighting this, we have added a paragraph that provides an overview of the five studies that were excluded from the pairwise meta-analysis for not directly reporting either clinical resolution or failure.

- Great they shared data and code…but I went to https://github.com/liliapsnh/cap_amoxicillin.git and got an error message. Same for https://github.com/Cherrylim128/AmoxForCap

  Our apologies about the link error. We have updated access for the github repository.

- Tau is a more direct measure of heterogeneity than I2. See the article by Rucker in BMC Med Res Methodol 8(1) p79. The problem with I2 is that it depends on the Ns. Try it yourself: take a

meta-analysis and look at I2. Now multiply all the Ns by 3, but keep all the effect sizes the same. See how I2 increased? Yeah. That's not good. Note that tau would not have increased, since it more purely measures the extent to which effect sizes differed.

Thank you for your note about this. We have replaced the I^2 statistics with tau, and updated the methodology accordingly.

- Curious about the risk of bias domain "selection of reported result" and on what basis some studies were penalized. Did they not report all the outcomes that their protocols said they'd collect? Or maybe their methods section mentioned more outcomes than their results sections provided? Did this have anything to do with them reporting clinical resolution or clinical failure?

  "Risk of bias in selection of the reported result" is one of the five domains of risk of bias for the Cochrane RoB2 tool. The questions guiding the bias result for this domain assess whether the result was produced in accordance with a pre-specified analysis plan that was finalized before unblinded outcome data was available for analysis. To directly assess this domain, firstly, the authors determined whether the results had been selected from multiple outcome measurements or analyses of the data. Secondly, the authors evaluated whether the trial was analysed in accordance with a pre-specified plan, such as reporting intention to treat results.

- I'm puzzled about the "Conclusion" sentence "Our findings provide a strong rationale for the WHO AWaRe book recommendation of using amoxicillin as a first-choice antibiotic for the treatment of CAP in adults and children". The main purpose of this work is not to investigate the effectiveness of amoxicillin itself, but rather to compare it to other antibiotics. So it looks odd to make that to focus of the overall "Conclusion".

  Thank you for your feedback on our conclusion. We have updated the conclusion of our main text to reflect the purpose of the work in comparing amoxicillin to other antibiotics. The conclusion now reads as follows:

  "In conclusion, we found no clear evidence of differences in clinical outcomes with amoxicillin compared to either amoxicillin–clavulanate or other antibiotics used for the treatment of CAP. While large uncertainties exist around the pooled estimates, the available evidence is consistent with the WHO AWaRe book recommendation to use amoxicillin as the first-choice antibiotic for the treatment of CAP in adults and children. Moreover, our findings emphasise the need for further randomised controlled trials comparing treatment options across populations and settings."

- Regarding fixed effects. Figure 5 is the one comparing amoxicillin amoxicillin to placebo and it used a fixed effects model even though I^2 was 67%. I would think random effects would be more appropriate, given that heterogeneity. The methods section said "We also evaluated the effect of amoxicillin against other antibiotic treatment on outcomes using fixed-effects logistic regression models as a sensitivity analyis, assuming there was a true underlying effect of amoxicillin on the outcome across the studies included in the meta-analysis." However the

justification for fixed effects should be not that we assume there IS an effect, but that all studies are measuring the same underlying MAGNITUDE of effect, which is a rarely made assumption.

Thank you for your feedback on the fixed-effects and random-effects models. Since a placebo is a comparator without issues of heterogeneity in dosage or frequency across the trials, we felt the assumption of a common magnitude of effect was worth considering in a sensitivity analysis in this case. Consequently, we focused on results from a fixed-effects model for those trials for a meaningful interpretation of the results as recommended by Peto R. 1987. Stat Med. PMID: 3616281. We have also revised the sentence in our methods section as below to clarify the underlying assumptions made:

"We used a fixed effects model for the trials comparing amoxicillin to a placebo, given that there was no variation in the dosage or frequency of the comparators across these trials."

VERSION 2 - REVIEW

**Reviewer**	**1**
**Name**	**Noma, Hisashi**
**Affiliation**	**The Institute of Statistical Mathematics, Department of Interdisciplinary Statistical Mathematics**
**Date**	**10-Mar-2026**
**COI**	

Thank you for the opportunity to review the revised version of this manuscript. I appreciate the authors' efforts in addressing the previous comments and improving the clarity of the methods and reporting. The manuscript has been strengthened and the additional analyses and explanations have improved the transparency of the study.

I have one remaining minor comment regarding the assessment of the transitivity assumption in the network meta-analysis.

Network meta-analysis relies on the transitivity assumption, meaning that the distribution of important effect modifiers should be sufficiently similar across treatment comparisons. In the revised manuscript, the authors indicate that they assessed transitivity by examining the distribution of age, setting, and dosage across studies. While this is helpful, guidance from the Cochrane Handbook for Systematic Reviews of Interventions and PRISMA-NMA recommends considering the broader set of potential effect modifiers that could influence treatment effects.

In the present network, additional factors may plausibly act as effect modifiers, such as disease severity of community-acquired pneumonia, comparator antibiotic class, and timing of outcome assessment. In particular, the comparator antibiotics vary substantially across the included studies (e.g., beta-lactams, macrolides, quinolones, sulfonamides), which could potentially influence the comparability of treatment effects across the network.

I therefore suggest that the authors provide a slightly more detailed description of how the plausibility of the transitivity assumption was evaluated. For example, a brief summary of the distribution of key potential effect modifiers across treatment comparisons, or a short clarification in the Methods or Discussion section, would further strengthen the methodological transparency of the network meta-analysis. The transitivity function implemented in the R NMA package (https://doi.org/10.1101%2F2025.09.15.25335823) may be helpful for exploring this issue.

VERSION 2 - AUTHOR RESPONSE

Reviewer: 1
Prof. Hisashi Noma, The Institute of Statistical Mathematics, The Graduate University for Advanced Studies
Comments to the Author:
Thank you for the opportunity to review the revised version of this manuscript. I appreciate the authors' efforts in addressing the previous comments and improving the clarity of the methods and reporting. The manuscript has been strengthened and the additional analyses and explanations have improved the transparency of the study.

I have one remaining minor comment regarding the assessment of the transitivity assumption in the network meta-analysis.

Network meta-analysis relies on the transitivity assumption, meaning that the distribution of important effect modifiers should be sufficiently similar across treatment comparisons. In the revised manuscript, the authors indicate that they assessed transitivity by examining the distribution of age, setting, and dosage across studies. While this is helpful, guidance from the Cochrane Handbook for Systematic Reviews of Interventions and PRISMA-NMA recommends considering the broader set of potential effect modifiers that could influence treatment effects.

In the present network, additional factors may plausibly act as effect modifiers, such as disease severity of community-acquired pneumonia, comparator antibiotic class, and timing of outcome assessment. In particular, the comparator antibiotics vary substantially across the included studies (e.g., beta-lactams, macrolides, quinolones, sulfonamides), which could potentially influence the comparability of treatment effects across the network.

I therefore suggest that the authors provide a slightly more detailed description of how the plausibility of the transitivity assumption was evaluated. For example, a brief summary of the distribution of key potential effect modifiers across treatment comparisons, or a short clarification in the Methods or Discussion section, would further strengthen the methodological transparency of the network meta-analysis. The transitivity function implemented in the R NMA package (https://doi.org/10.1101%2F2025.09.15.25335823) may be helpful for exploring this issue.

Thank you for your feedback and insights about the transitivity assumption. We have added the distribution of the severity of community-acquired pneumonia and timing of outcome to the supplementary information (Supplementary Table 3). We have also updated the main text (methods and discussion sections) to reflect these changes and provide a brief summary of the distribution of these key potential effect modifiers across treatment comparisons.

This methods section now reads:
"Finally, we conducted an unplanned frequentist random-effects network meta-analysis to compare amoxicillin and amoxicillin–clavulanate, assuming that each study had its own true effect. Network meta-analysis relies on the transitivity assumption—that the distribution of effect modifiers is similar across treatment comparisons. To mitigate the risk of violating this assumption, we restricted the analysis to trials involving adult patients only. Furthermore, we assessed the transitivity assumption by examining the distribution of potential effect modifiers—age, setting, dosage, duration, and CAP severity—across treatment comparisons.
These potential effect modifiers were chosen to ensure there were no significant differences that could influence the comparability of treatment effects across the network. In addition, net heat plots and the node-splitting method were used to evaluate agreement between direct and indirect evidence in the network (i.e., the consistency assumption). The netmeta package was used for the network plots and network meta-analysis."

And the results section now reads:
"The global Q score for inconsistency was 7.36 (p-value=0.195), and the only comparisons that were inconsistent were ceftriaxone versus amoxicillin (p-value 0.021) and ceftriaxone versus amoxicillin-clavulanate (p-value 0.018) (Supplementary Table 2, Supplementary Figure 17).
However, the tests have limited power as the network of the 28 studies was sparse. We found no clear evidence of violations of the transitivity assumption when comparing age, setting, dosage, duration, and severity of studies across the comparisons (Supplementary Table 3). The mean age across all amoxicillin or amoxicillin-clavulanate arms for the 28 studies was 51.5 years with a standard deviation of 7.83 and the average duration was approximately 8.96 years with a standard deviation of 1.94 years. However, the number of studies per comparison was small."

VERSION 3 - REVIEW

**Reviewer**	**1**
**Name**	**Noma, Hisashi**

Affiliation The Institute of Statistical Mathematics, Department of Interdisciplinary Statistical Mathematics

Date 06-Apr-2026

COI

I appreciate the authors' efforts in addressing the previous comments and improving the clarity and transparency of the methodological description.

In particular, the additional information provided on the distribution of key potential effect modifiers, including disease severity and timing of outcome assessment, as well as the corresponding revisions to the Methods and Supplementary Materials, have strengthened the assessment of the transitivity assumption. These additions improve the overall interpretability of the network meta-analysis.

I note that some potential concerns remain regarding the heterogeneity of comparator antibiotic classes across studies, which could, in principle, act as effect modifiers. While this aspect could have been explored in more depth, I consider that the authors have made a reasonable and proportionate effort to evaluate the plausibility of the transitivity assumption within the constraints of the available data.
